# Defining T Cell Subsets in Human Tonsils Using ChipCytometry

**DOI:** 10.4049/jimmunol.2100063

**Published:** 2021-06-15

**Authors:** Joachim P. Hagel, Kyle Bennett, Francesca Buffa, Paul Klenerman, Christian B. Willberg, Kate Powell

**Affiliations:** * Peter Medawar Building for Pathogen Research, Nuffield Department of Medicine, University of Oxford, Oxford, United Kingdom;; † Computational Biology and Integrative Genomics, Department of Oncology, University of Oxford, Oxford, United Kingdom;; ‡ NIHR Biomedical Research Centre, John Radcliffe Hospital, Oxford University Hospitals NHS Foundation Trust, Oxford, United Kingdom; and; § Translational Gastroenterology Unit, Nuffield Department of Medicine, University of Oxford, Oxford, United Kingdom

## Abstract

ChipCytometry is a multiplex imaging method that can be used to analyze either cell suspensions or tissue sections. Images are acquired by iterative cycles of immunostaining with fluorescently labeled Abs, followed by photobleaching, which allows the accumulation of multiple markers on a single sample. In this study, we explored the feasibility of using ChipCytometry to identify and phenotype cell subsets, including rare cell types, using a combination of tissue sections and single-cell suspensions. Using ChipCytometry of tissue sections, we successfully demonstrated the architecture of human palatine tonsils, including the B and T cell zones, and characterized subcompartments such as the B cell mantle and germinal center zone, as well as intrafollicular PD1-expressing CD4^+^ T cells. Additionally, we were able to identify the rare tonsillar T cell subsets, mucosal-associated invariant T (MAIT) and γδ-T cells, within tonsil tissue. Using single-cell suspension ChipCytometry, we further dissected human tonsillar T cell subsets via unsupervised clustering analysis as well as supervised traditional manual gating. We were able to show that PD1^+^CD4^+^ T cells are comprised of CXCR5^+^BCL6^high^ follicular Th cells and CXCR5^−^BCL6^mid^ pre–follicular Th cells. Both supervised and unsupervised analysis approaches identified MAIT cells in single-cell suspensions, confirming a phenotype similar to that of blood-derived MAIT cells. In this study, we demonstrate that ChipCytometry is a viable method for single-cell suspension cytometry and analysis, with the additional benefit of allowing phenotyping in a spatial context using tissue sections.

## Key Points

- ChipCytometry was used for multiplex immunophenotyping of human tonsils.
- It is viable for high-content profiling of cell suspensions and tissue sections.
- ChipCytometry can identify and localize cell populations and rare cell types.

## Introduction

Many novel technologies have recently emerged for multiparameter cytometry analysis of cells and tissues, offering the exciting prospect of visualizing cellular phenotyping within a spatial context. These methods use a variety of immunostaining methodologies applied to fluorescence microscopy or mass spectrometry imaging techniques to acquire information on the expression of multiple markers. Each method has its own advantages and disadvantages. For example, the number of markers that can be assessed on a single sample, the sample processing time and throughput, the commercial availability of reagents, operating costs, and system purchasing costs are system specific (1–5).

ChipCytometry is an imaging-based multiplex-immunophenotyping method in which samples, either cell suspensions or tissue sections, are immobilized on slides inside a microfluidic chip (6). Through consecutive and iterative cycles of staining with fluorophore-labeled Abs, imaging and subsequent photobleaching, a theoretically unlimited number of markers can be measured on a single sample. Advantages of ChipCytometry over other methods include that samples are preserved after analysis and can be stored for at least 20 mo without significant degradation (7). This allows reanalysis with additional markers or sample collection and storage at different sites with subsequent centralized analysis. In addition, the development of a panel of markers is relatively straightforward using standard flow cytometry Abs. Combinations of up to five fluorophores can be used for each cycle.

To explore the feasibility of using ChipCytometry for immune-phenotyping, we employed lymphoid palatine tonsil tissue, as it is very rich in immune cells and has a well-established and distinct architecture. Palatine tonsils are secondary lymphoid tissues that belong to the Waldeyer’s ring, which further includes the adenoids and tubal and lingual tonsils (8, 9). They are located at the transition between the mouth and oropharynx and are a part of the mucosal-associated lymphoid tissues (10, 11). The tonsils are covered by an epithelium that increases the tonsil surface by forming crypts in which specialized cells can take up Ag and transport it to subepithelial areas where it can get in contact with immune cells (10, 12). The immune cells are organized in two major zones within the tonsils: B cell follicles and the extrafollicular region in which T cells and B cells become activated. Lymphoid follicles contain B cells and are therefore characterized by CD19 expression. Primary lymphoid follicles do not have a germinal center (13). Secondary lymphoid follicles consist of a mantle zone including naive (IgD^+^CD10^−^) B cells; they also contain a germinal center including germinal center (IgD^−^CD10^+^) B cells (14, 15). Germinal centers are places of B cell proliferation, differentiation, and maturation (13). Besides B cells, lymphoid follicles also contain a network of stromal-derived follicular dendritic cells, which are important for Ag presentation to B cells (16). Additionally, B cell follicles contain T cells that are critical for the humoral immune response (17). The extrafollicular region (also called the T cell zone) contains T cells that are primarily CD4^+^ T cells with fewer CD8^+^ T cells. Besides T cells, the extrafollicular region includes interdigitating dendritic cells, macrophages, and high-endothelial venules. The latter are specialized venules that allow circulating lymphocytes to enter the lymphoid tissue (13).

Tonsillar CD4^+^ T cell subsets can be divided into three major groups (18). Most intrafollicular T cells belong to the follicular B helper T cell group (follicular Th [TFH], CD4^+^PD1^+^CXCR5^+^BCL6^high^) (17, 18). B cell and TFH interactions are crucial for the formation of the humoral immune response and support B cell somatic hypermutation and Ab class switching (17, 19). Other tonsillar CD4^+^ T cell subsets include non-TFH (CD4^+^PD1^−^CXCR5^−^BCL6^low^) and pre-TFH (CD4^+^PD1^+^CXCR5^−^BCL6^mid^). Pre-TFH cells develop from activated Ag-specific CD4^+^ T cells. The transcriptional program that is initiated in pre-TFH cells can induce CXCR5 expression, enabling the cells to migrate toward the B cell follicle (18, 20).

Most CD4^+^ and CD8^+^ T cells that enter palatine tonsils are naive cells competing for Ag to reach maturity (10, 13), but there are also unconventional T cell subsets found within the tonsil, for example, innate-like T cells including mucosal-associated invariant T (MAIT) cells and γδ-T cells (21–23). MAIT cells are memory-type and Ag-experienced cells with peripheral tissue homing properties (24, 25). They are characterized by their semi-invariant TCR that recognizes microbial-derived riboflavin metabolites presented by a conserved MHC class I–like molecule called MR1 (26, 27). MAIT cells can, however, also be activated in a TCR-independent manner by cytokines such as IL-12, IL-18, and IL-15 (28, 29). Most MAIT cells are CD8^+^ or double negative (DN) in blood. They can be identified by tetramer staining or using a combination of markers including Vα7.2 and CD161 (30, 31). MAIT cells also express the innate-like marker CD218a (IL-18Rα) and the transcription factor PLZF (31). MAIT cell selection and development begin in the thymus, and MAIT cells are thought to acquire their memory Ag-experienced phenotype via contact with microbes in the periphery (32). MAIT cell frequencies in tonsil are ∼10 times lower than in blood (21). MAIT cell responses play multiple roles in infection, inflammation, and cancer (29, 33, 34).

To establish the utility of the ChipCytometry technique, using both cell suspensions and tissue sections, we aimed to depict the general tonsil architecture, showing different tonsillar regions and subcompartments. We localized and phenotyped immune subsets including follicular CD4^+^ T cell subsets and rarer immune cells such as MAIT cells and finally analyzed data obtained from ChipCytometry of cell suspensions for more stringent phenotyping of immune subsets.

## Materials and Methods

### Tissues and cells

Human tonsillar tissue was obtained from routine tonsillectomies collected by the Translational Gastroenterology Unit Biobank, John Radcliffe Hospital (Oxford, U.K.) following informed consent under approved study protocol 16/YH/O247. Tissue sections were prepared by snap-freezing samples in OCT cutting medium (OCT embedding matrix; CellPath) and cryosectioning onto glass coverslips. Single-cell suspensions were prepared by mechanical disruption of tonsil samples. Human blood samples were obtained from the National Health Service Blood and Transplant, John Radcliffe Hospital. PBMCs were isolated from on a Lymphoprep gradient (STEMCELL Technologies) according to the manufacturer’s instructions.

### ChipCytometry of cell suspensions

Tonsil-derived CD3^+^ T cells were isolated by positive selection from single-cell suspensions using magnetic CD3 Microbeads (Miltenyi Biotec) and treated with Human TruStain FcX (BioLegend) to block unwanted Fc receptor–mediated staining. Cells from different donors were barcoded with anti-CD45 Abs (pan–leukocyte marker) coupled to FITC, PE, or PerCP-Cy5.5 to allow analysis of multiple donors on a single chip. Barcodes consisted of different single anti-CD45 stains or combinations of differently labeled anti-CD45 Abs. The CXCR5 Ab does not recognize fixed epitope, and therefore, staining was performed simultaneously with CD45 barcode staining for 5 min at 4°C. Donor-derived cells were mixed and loaded into cell suspension chips (ZellSafe Cells – Chips; Zellkraftwerk, Leipzig, Germany), allowed to adhere to the chip surface for 10 min at room temperature, then nonadhered cells were washed off with PBS. The initial acquisition, performed with the ChipCytometer (Zellscanner ONE; Zellkraftwerk), included barcodes and CXCR5 stain. Samples were fixed (fixation buffer; Zellkraftwerk) for 5 min at room temperature, and subsequent markers were acquired in iterative rounds of photobleaching, staining, and imaging. FITC-, PE-, or PerCP/PerCP-Cy5.5–coupled Abs were applied in mixes (up to three per round). Surface Abs were incubated for 10 min at room temperature. For intracellular markers, True-Nuclear Transcription Factor Buffer Set (BioLegend) was used as follows: chips were rinsed and incubated with permeabilization buffer for 1 h, followed by rinsing and incubation with fixation buffer for 1 further h before finally washing with permeabilization buffer. Ab mixes for intracellular staining were prepared in permeabilization buffer and incubated for 30 min (all permeabilization and staining steps performed at room temperature). The following 19 markers were applied to the samples: BCL6, CD3, CD4, CD8, CD19, CD44, CD45, CD45RA, CD56, CD69, CD161, CXCR5, FASL, FOXP3, Granzyme B (GZMB), ICOS, IL-18Rα (CD218a), PD1, and Vα7.2. For Ab details, please see Tables I and II.

**Table I.** Extracellular markers for ChipCytometry cell suspension staining

	Marker	Clone	Label	Manufacturer
1	CD3	UCHT 1	PerCP-Cy5.5	BioLegend
2	CD4	RPA-T4	FITC	BioLegend
3	CD8α	SK1	PerCP-Cy5.5	BioLegend
4	CD19	LT19	PE	Miltenyi Biotec
5	CD44	DB105	PE	Miltenyi Biotec
6	CD45	HI30	FITC, PE, PerCP	BioLegend
7	CD45RA	HI100	PerCP-Cy5.5	BioLegend
8	CD56*	AF12-7H3	PE	Miltenyi Biotec
9	CD69	FN50	PE	BioLegend
10	CD161	191B8	PE	Miltenyi Biotec
11	CD178 (FASL)*	NOK-1	PE	BioLegend
12	CD185 (CXCR5)	J252D4	BV421	BioLegend
13	CD218a (IL-18Ra)*	H44	PE	BioLegend, eBioscience
14	CD278 (ICOS)	C398.4A	PerCP-Cy5.5	BioLegend
15	CD279 (PD-1)	eBioJ105	PE	eBioscience
16	TCR Vα7.2	3C10	PE	BioLegend

Markers that are sensitive toward repeated cycles of photobleaching are indicated with an asterisk and should be stained early in the process.

**Table II.** Intracellular markers for ChipCytometry cell suspension staining

	Marker	Clone	Label	Manufacturer
1	BCL6	REA373	PE	Miltenyi Biotec
2	GZMB	GB11	PE	eBioscience
3	FOXP3	259D/C7	PE	BD Biosciences

### ChipCytometry of tissue sections

Cryosections were cut directly onto poly-l-lysine– or APES-coated coverslips (Sigma-Aldrich) and fixed immediately using freshly prepared 0.1 M phosphate-buffered 4% paraformaldehyde (Sigma-Aldrich) or Zellkraftwerk fixation buffer (Zellkraftwerk) for 10 min at room temperature. After washing in PBS, sections on coverslips were assembled into tissue chips (ZellSafe Tissue – Chips; Zellkraftwerk), which were immediately filled with PBS or storage buffer (Zellkraftwerk). Before use, the storage buffer was washed out of the chip with ∼5 ml of PBS. Tonsil sections were initially blocked by incubating in 3% normal goat serum with 2% BSA (Thermo Fisher Scientific) in PBS for at least 1 h at room temperature. Immunostaining was performed at room temperature for 30 min using 0.5 ml of diluted Ab solution per chip. Ab mixtures were diluted in PBS alone or PBS containing 2% BSA or 2% normal goat serum. A total of 12 markers were acquired in iterative rounds of photobleaching, staining, and imaging: CD3, CD4, CD8, CD10, CD19, CD161, γδTCR, IgD, PD1, PLZF, Vα7.2, and histone H3 (not shown). For Ab details please see Table III.

**Table III.** Markers for ChipCytometry tissue section staining

	Marker	Clone	Label	Manufacturer
1	CD3	UCHT1	PerCP	BioLegend
2	CD4	RPA-T4	PerCP	BioLegend
3	CD8	SK1	FITC	BioLegend
4	CD10	HI10a	PE	BioLegend
5	CD19	LT19	PerCP	ExBio
6	CD161	HP-3G10	PE	eBioscience
7	CD279 (PD-1)	eBioJ105	PE	eBioscience
8	IgD	IA6-2	PerCP	BioLegend
9	Histone H3	17H2L9	AF488	Invitrogen
10	PLZF	R17-809	PE	BD Pharmingen
11	TCR-γ/δ (γδTCR)	5A6.E9	FITC, PE	Life Technologies
12	TCR Vα7.2	3C10	PE	BioLegend

AF488, Alexa Fluor 488.

### Flow cytometry

Cells were stained with a viability dye (LIVE/DEAD Fixable Near-IR Dead Cell Stain Kit; Invitrogen) and fluorochrome-labeled Abs for 30 min at 4°C. The following Abs were used: CD3–eFluor 450 (OKT3; eBioscience), CD8-VioGreen (BW135/80; Miltenyi Biotec), γδTCR-FITC (5A6.E9; Invitrogen), CD161-PE and CD161-APC (191B8; Miltenyi Biotec), Vδ2-PerCP-Cy5.5 (B6; BioLegend), CD56–PE-Cy7 (HCD56; BioLegend), Vα7.2-APC and Vα7.2-PerCP-Cy5.5 (3C10; BioLegend), CCR7-FITC (G043H7), and CD62L-PE (DREG-56; BioLegend). Data were acquired by a MACSQuant Analyzer 10 (Miltenyi Biotec).

### Data processing and statistical methods

Flow cytometry and ChipCytometry data were analyzed using FlowJo (versions 10.6.2 and 10.7.1; Beckton Dickinson), and bar charts were generated using GraphPad Prism software (version 8.4.3). For supervised manual gating analysis of flow cytometry and ChipCytometry data, biexponential transformation was applied in FlowJo. For unsupervised clustering analysis, ChipCytometry cell suspension data were first transformed and preprocessed in FlowJo and then exported to R (RStudio version 1.2.5042 and R version 3.6.2). The data were transformed using HyperLog transformation. The parameters of this transformation were adjusted, and rescaling was performed so that for each marker, the distribution spanned the whole dynamic range. The transformed data were binned into integer histogram channels ranging from 0 to 1023. Outliers and fluorescence anomalies outside the axis limits after transformation were assigned to either the minimum or maximum value. All datapoints with an assigned value of 0 or 1023 after the transformation were removed from further analysis. An exception was made for CXCR5, which was transformed using logarithmic transformation, because suitable rescaling could not be achieved with HyperLog in FlowJo. Data were preprocessed by excluding CD19^+^ cells and gating on CD3^+^ cells in FlowJo. FlowSOM R package (version 1.18.0) (35) was used to identify cell clusters within the dataset using all markers except for CD45, which was used for barcoding, and CD3 and CD19, as these were used for gating during preprocessing. Parameters chosen were self-organizing map (SOM) grid = 10 × 10 and seed = 1234. The resulting SOM nodes were used in the R package ConsensusClusterPlus (1.50.0) to obtain the final optimal clusters (36). A delta area plot was created with a maximum of *k* = 20 metaclusters, which indicated cluster stability at a metacluster number of *k* = 12. Cluster information for 12 metaclusters was exported, and data were further analyzed in FlowJo for visualization, including *t*-distributed stochastic neighbor embedding (*t*-SNE) analysis (fast Fourier transform–accelerated interpolation-based *t*-SNE, iteration: 3000, perplexity: 50, *k*-nearest neighbors algorithm: exact, and learning rate: auto [optimized *t*-SNE]). For cluster characterization, the distribution of the intensity of each marker was considered in each cluster and the median value taken (median marker expression). This was displayed as heatmap using pheatmap package (version 1.0.12). In addition to comparing median marker expression directly, Marker Enrichment Modeling was performed for cluster characterization using the R package Marker Enrichment Modeling (version 2.0.0) (37, 38).

### Image processing

Images for publication were downloaded from the ChipCytometer as 16-bit uncompressed tagged image file format (TIFF) files and converted to 8-bit per channel RGB TIFF files in ImageJ/Fiji (version 1.5). Scale bars were added in Adobe Photoshop CS4 according to the metadata stored with the image. Images were cropped, annotations (arrows, text, etc.) were added, and brightness and contrast adjusted as necessary in Photoshop. Files were exported as flattened 300 dpi TIFF files with Lempel–Ziv–Welch compression. Files of each image processing stage were saved.

## Results

### Defining tonsillar architecture in humans using tissue ChipCytometry

**FIGURE 1.**
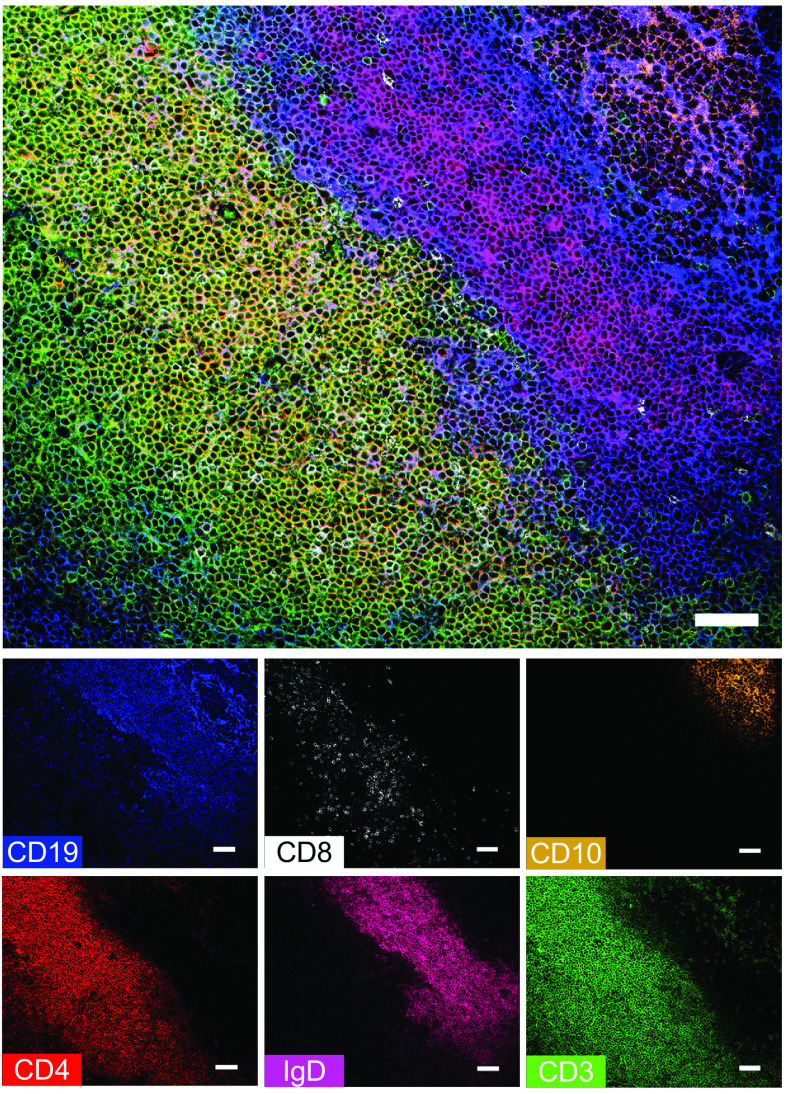
Human tonsil architecture resolved using ChipCytometry. Sections of paraformaldehyde-fixed human tonsil tissue mounted in microfluidic chips were immunostained in consecutive runs using a panel of 12 fluorochrome-labeled Abs. The top image was compiled by selecting six markers: CD19 (blue), CD8 (white), CD10 (orange), CD4 (red), IgD (magenta), and CD3 (green), assigning colors arbitrarily from the lookup table and merging to form a composite image. Expression of individual markers is shown in the small panels. The B cell zone (B cell follicle) is characterized by CD19 expression, in which IgD outlines the mantle zone containing IgD-expressing naive B cells, and CD10 outlines the germinal center containing CD10^+^ germinal center B cells. Adjacent to the B cell zone, CD3 expression outlines the T cell zone (extrafollicular zone), including CD4^+^ and CD8^+^ T cells. Scale bars, 50 µm.

A panel of 12 Abs was employed to establish the anatomy of the palatine tonsil by ChipCytometry. A combination of T cell markers (CD3, CD4, and CD8) and B cell markers (CD19, CD10, and IgD) were chosen to depict general palatine tonsil architecture (Fig. 1, Supplemental Figs. 1A, 2A). Specifically, the lymphoid follicle zone (B cell zone) was defined by CD19 expression and could be further characterized by the differential expression of IgD and CD10. Naive B cells (IgD^+^CD10^−^) are mainly located in the B cell mantle zone, and germinal center B cells (IgD^−^CD10^+^) are located in the B cell germinal center zone (14, 15). The extrafollicular zone (T cell zone) adjacent to the B cell zone was defined by CD3 expression. CD3^+^ T cells included CD4-expressing and CD8-expressing cells. A few CD3^+^ T cells could also be found within the B cell zone.

### Identification of CD4 T cell subsets in human tonsils

**FIGURE 2.**
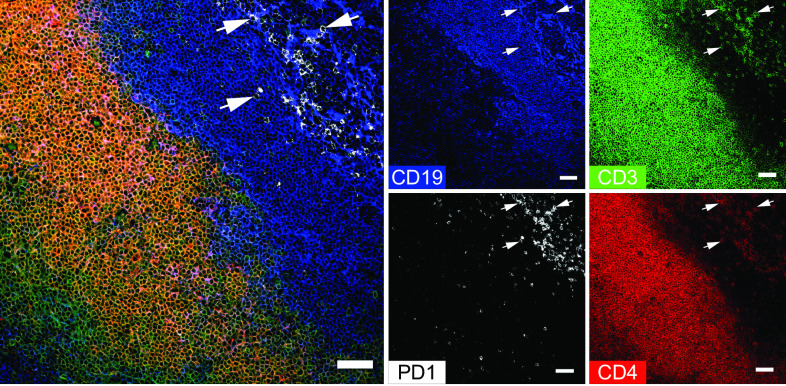
Identification of CD4^+^ T cell subsets in human tonsils. A combination of CD4, CD19, CD3, and PD1 were chosen from the full marker panel to identify CD4^+^ T cells in the tonsil. The large multicolor composite image shows a merged image of CD19 (blue), CD3 (green), PD1 (white), and CD4 (red), whereas the smaller images demonstrate the expression of each marker individually. The T cell zone is characterized by CD3 expression and includes CD4^+^ T cells; CD3^+^CD4^+^ cells appear yellow/orange in combination. PD1-expressing CD3^+^CD4^+^ are predominantly located in the B cell zone, but a few are also located in the T cell zone. This subset most likely includes prefollicular (pre-TFH) and TFH cells. CD3^+^CD4^+^PD1^−^ cells are predominantly located in the extrafollicular region and include non-TFH cells. Arrowheads indicate examples of CD3^+^CD4^+^PD1^+^ cells. Scale bars, 50 µm.

From the full panel of 12 markers, a combination of CD3, CD4, PD1, and CD19 staining was chosen to identify palatine tonsillar T cell subsets expressing PD1 (Fig. 2, Supplemental Figs. 1B, 2B). CD3 expression was concentrated within the extrafollicular T cell zone; however, we were also able to identify some intrafollicular CD3^+^ T cells. Many of those T cells were CD4^+^ and PD1^+^. A few rare CD4^+^PD1^+^ T cells could also be found within the extrafollicular region (T cell zone). Tonsillar CD3^+^CD4^+^PD1^+^ T cells most likely include pre-TFH and TFH subsets (18) as opposed to CD3^+^CD4^+^PD1^−^ cells that most likely include non-TFH cells. Examples of some of the many CD3^+^CD4^+^PD1^+^ T cells are indicated in (Fig. 2 and Supplemental Fig. 1B by white arrowheads.

### Identification and phenotyping of tonsillar CD3 T cell subsets by cell suspension ChipCytometry

**FIGURE 3.**
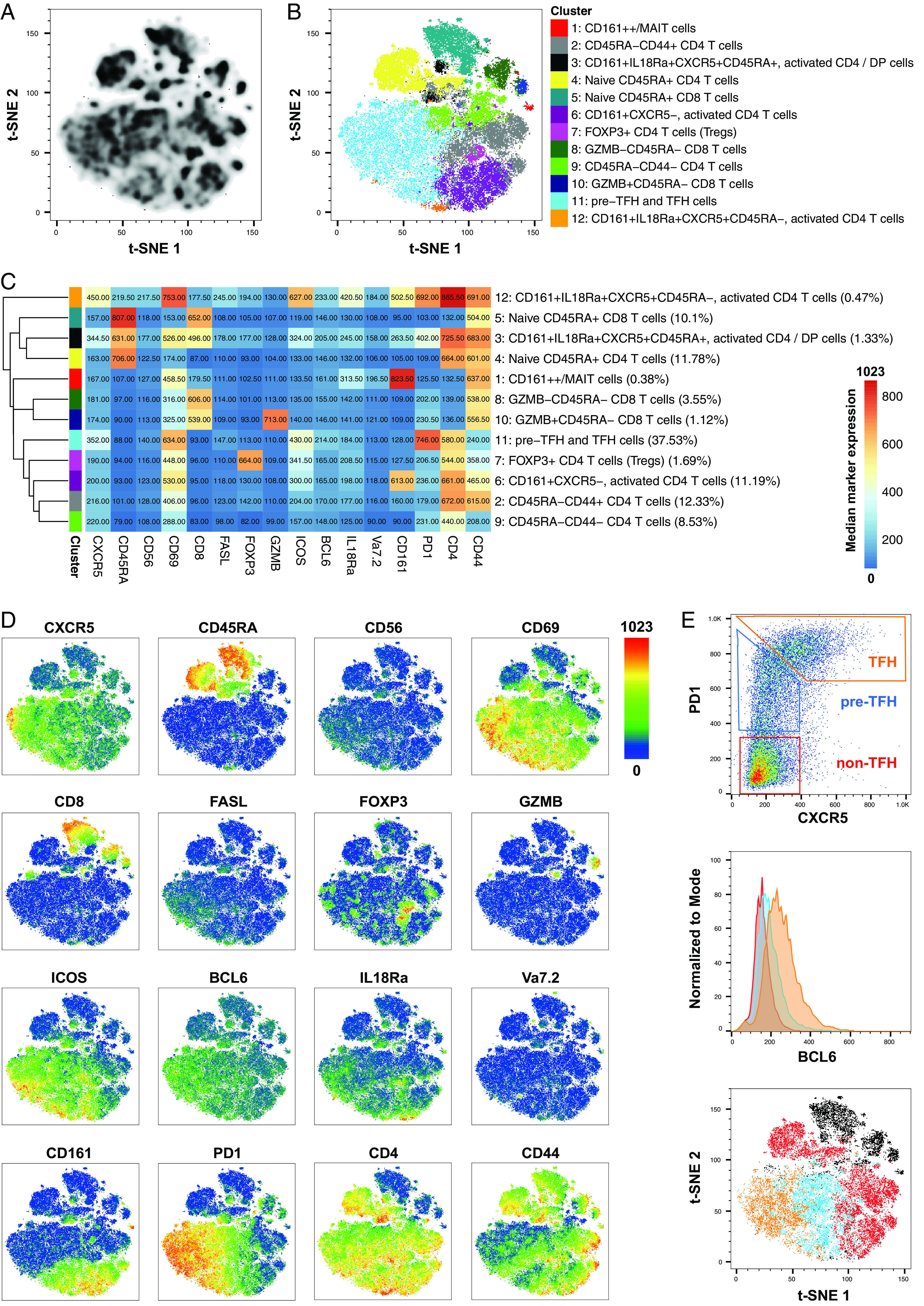
Phenotyping of tonsil-derived, CD3-enriched cells by cell suspension ChipCytometry. Four human tonsils were processed to single-cell suspensions, positively enriched for CD3^+^ T cells, barcoded, loaded onto a microfluidic chip, fixed, and subsequently analyzed by ChipCytometry with a panel of 19 fluorochrome-labeled Abs. (**A**) Density plot shows *t*-SNE analysis of concatenated samples, including all CD3^+^CD19^−^ cells. (**B**) Clusters that were identified via FlowSOM were projected onto the *t*-SNE coordinates and color coded as indicated. (**C**) A heatmap showing median marker intensities within each cell cluster was generated to characterize cell clusters and derive cell labels. The heatmap color represents median marker expression across all four aggregated samples. (**D**) T-SNE plots show the expression of 16 markers within the aggregated samples. (**E**) Top panel, Within CD4^+^ T cells, the subsets TFH, pre-TFH, and non-TFH cells were identified according to expression of CXCR5 and PD1. Center panel, BCL6 expression is shown within non-TFH (red), pre-TFH (blue), and TFH (orange) cell subsets in human tonsils. Bottom panel, T-SNE plot shows non-TFH (red), pre-TFH (blue), TFH (orange), and non-CD4 T cells (black). Plots in (E) are derived from one donor and are representative of four donors.

To identify and phenotype tonsillar CD3 T cell subsets, cell suspensions produced by dissociation of human palatine tonsil tissue were enriched for CD3^+^ T cells (*n* = 4). Cells from each donor were barcoded with CD45 Abs coupled to different fluorochromes, allowing individual donors to be distinguished in the pooled samples. A staining panel consisting of the following 19 markers was used: BCL6, CD3, CD4, CD8, CD19, CD44, CD45, CD45RA, CD56, CD69, CD161, CXCR5, FASL, FOXP3, GZMB, ICOS, IL-18Rα (CD218a), PD1, and Vα7.2. T cell subsets were identified via unsupervised clustering using FlowSOM, a method based on SOM clustering (35, 39), and visualized using *t*-SNE (Fig. 3A). ConsensusClusterPlus was used to estimate the optimal number of clusters within the dataset, and FlowSOM clustering was performed into 12 groups (Fig. 3B). As the process for labeling cell clusters is not established, we applied a layered approach for cluster labeling using median marker expression together with Marker Enrichment Modeling in a semisupervised manner in addition to gating in FlowJo (Fig. 3C, Supplemental Fig. 3A, 3B). The identified CD3^+^ T cell subsets included various CD4^+^ T cell subsets, CD8^+^ T cell subsets, and unconventional, innate-like T cell subsets such as MAIT cells. We found one naive CD4^+^CD45RA^+^ T cell subset (cluster 4) and two CD4^+^CD45RA^−^ subsets that differed in regard to CD44 expression (cluster 2 = CD44^+^ and cluster 9 = CD44^−^). Each of these subsets comprised ∼10% of total T cells. Additionally, we found three subsets that contained CD4^+^ T cells expressing CD161, which is a marker generally associated with innate-like and memory cell types (40). Those were CD161^+^CXCR5^−^CD69^+^ICOS^+^CD45RA^−^ (cluster 6, ∼10% of total T cells), CD161^+^CXCR5^+^IL-18Rα^+^CD69^+^PD1^+^ICOS^+^CD45RA^−^ (cluster 12, 0.47% of total T cells), and CD161^+^CXCR5^+^IL-18Rα^+^CD69^+^PD1^+^ICOS^+^CD45RA^+^ (cluster 3, 1.33% of total T cells). The latter included, besides CD4^+^ T cells, a substantial fraction of double-positive T cells and showed lower levels of CD161, CXCR5, IL-18Rα, CD69, PD1, and ICOS expression compared with cluster 12. Other CD4^+^ T cell subsets included FOXP3^+^ cells, which represent the T regulatory cell population (cluster 7, 1.69% of total T cells) and prefollicular/follicular CD4 Th cells (cluster 11, CD4^+^CXCR5^+/−^PD1^+^ICOS^+^CD69^+^), which comprised 37.53% of total T cells. In addition to CD4 T cell subsets, we found a naive CD8^+^CD45RA^+^ subset (cluster 5, ∼10% of total T cells) and two CD8^+^CD45RA^−^ subsets, of which one was GZMB^+^ (cluster 10, 1.12% of total T cells), and the other was GZMB^−^ (cluster 8, 3.55% of total T cells), respectively. We also found a cluster that contained CD161^++^ T cells, including MAIT cells (CD161^++^Vα7.2^+/−^IL-18Rα^+^CD69^+^, cluster 1, 0.38% of total T cells), which were either CD8^+^ or DN. The expression of the 16 phenotyping markers that were used for clustering are displayed on *t*-SNE plots in (Fig. 3D.

We further analyzed the dataset via manual gating to elaborate on the characterization of PD1-expressing and follicular CD4 T cell subsets. We stratified CD4^+^ T cells according to PD1 and CXCR5 expression (Fig. 3E), as previously shown by Thornhill et al. (18). The subsets included CXCR5^+^PD1^+^ TFH, CXCR5^−^PD1^+^ pre-TFH, and CXCR5^−^PD1^−^ non-TFH cells. These cell types differed in BCL6 expression; TFH cells had the highest levels of BCL6 expression, pre-TFH intermediate levels of BCL6 expression, and non-TFH the lowest levels of BCL6 expression. These three CD4 subsets also segregated on the *t*-SNE plot. With our characterization of tonsil-derived CD4 T cell subsets, we were able to replicate results previously described in the literature by Thornhill et al. (18).

### Identifying rarer T cell subsets in tonsils by tissue ChipCytometry including MAIT cells and γδ-T cells

**FIGURE 4.**
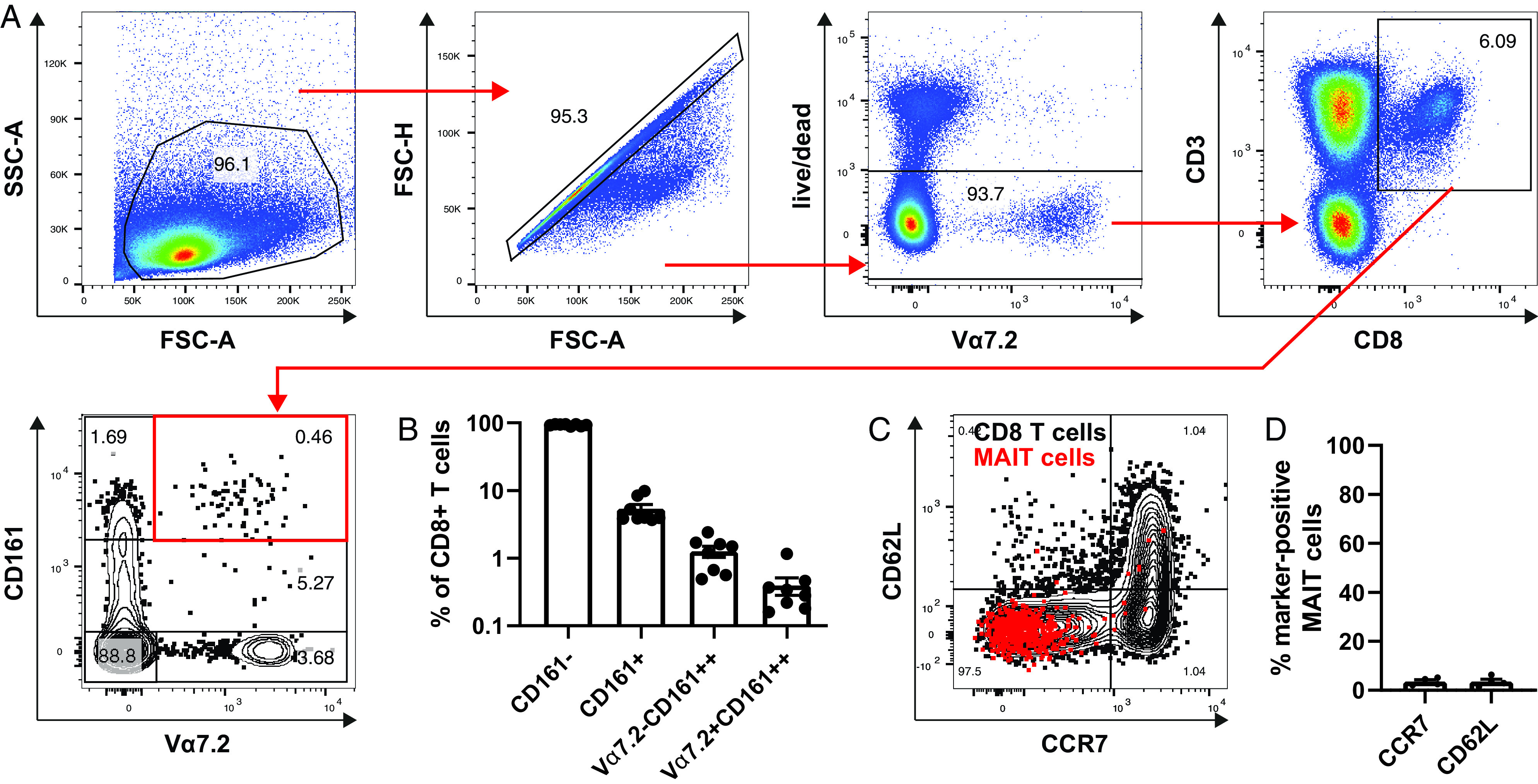
Flow cytometry identifies CD8^+^ MAIT cells as a rare subset within human tonsils. Human tonsils were processed to single-cell suspensions and analyzed by flow cytometry. Stains included LIVE/DEAD Fixable dye, CD3, CD8, Vα7.2, CD161, CCR7, and CD62L. (**A**) Gating strategy is shown to identify CD8^+^ MAIT cells (Vα7.2^+^CD161^++^) within tonsils. (**B**) Enumeration of CD8 T cell subsets (eight tonsils from six donors). (**C**) CCR7 and CD62 expression of total CD8 T cells (black) and MAIT cells (red). The flow cytometry plot is representative of four biological replicates. (**D**) Enumeration of CCR7 and CD62L expressing MAIT cells (*n* = 4).

Next, we aimed to identify and phenotype rare cell subsets within palatine tonsillar tissue. MAIT cells have been described as a rare cell subset within human palatine tonsil tissue (21, 22). We could confirm this by the analysis of eight palatine tonsils of six different donors using flow cytometry (Fig. 4A, 4B). MAIT cells were defined as CD3^+^CD8^+^Vα7.2^+^CD161^++^. The frequency of MAIT cells ranged from ∼0.1 to 1% of total CD8 T cells. This is 10-fold lower compared with blood (41). Interestingly, palatine tonsillar MAIT cells did not show typical lymphoid tissue homing marker expression CCR7 and CD62L (Fig. 4C, 4D).

**FIGURE 5.**
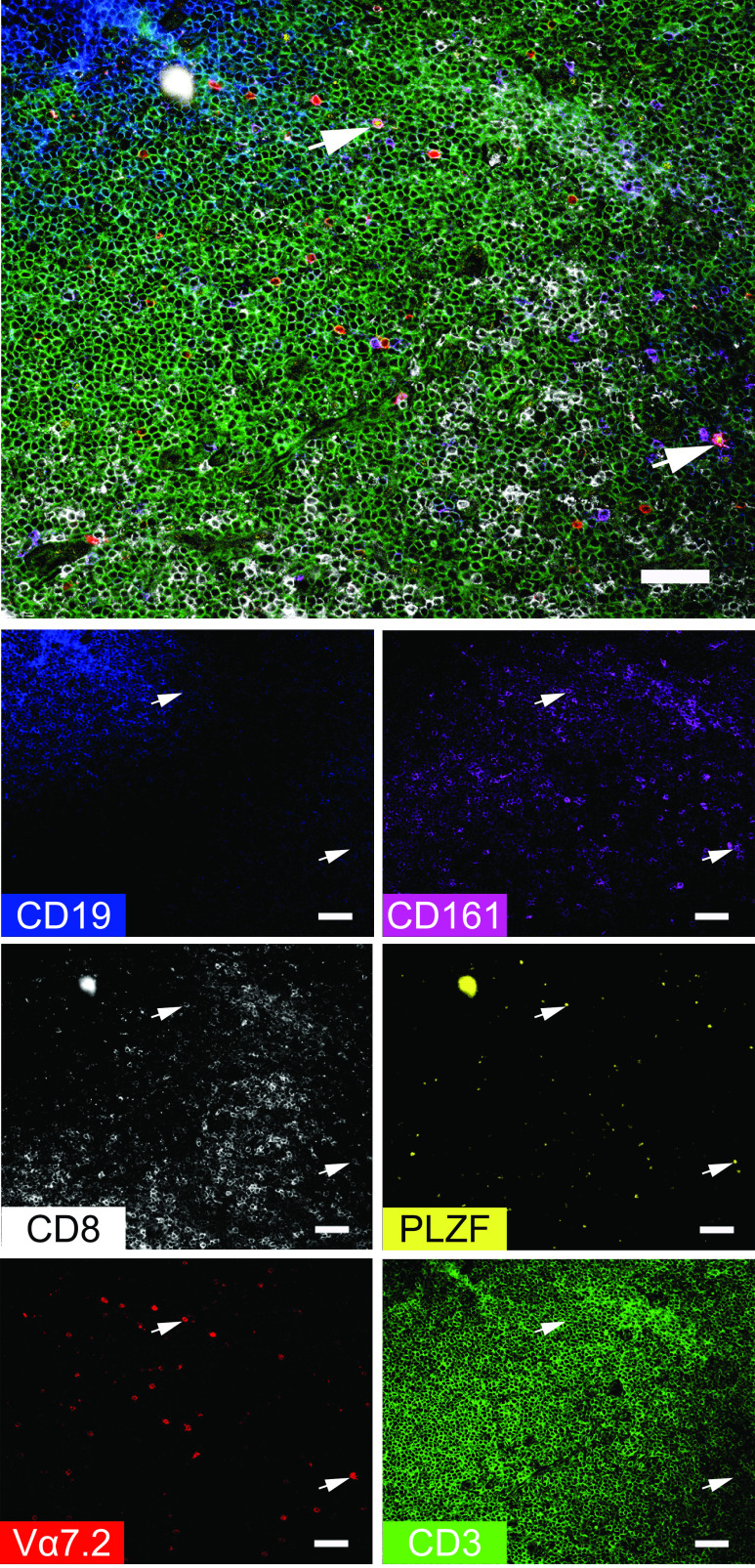
Identification of rare immune subsets in human tonsils. A combination of Vα7.2 (shown in red), CD8 (white), CD161 (magenta), PLZF (yellow), CD3 (green), and CD19 (blue) markers were selected from the available panel to illustrate the presence of MAIT cells within the tonsil. Single-color images demonstrate the expression of each marker individually, whereas the larger image shows a merged composite of all six markers. Arrowheads indicate examples of cells that coexpress CD3, CD8, Vα7.2, CD161, and PLZF, which represent CD8^+^ MAIT cells. During the iterative staining process, dust particles can be introduced into the microfluidic chip, generating autofluorescent artifacts such as those seen in this study at the top left of the CD8, PLZF, and merged images. Scale bars, 50 µm.

We were able to identify CD8^+^ MAIT cells in palatine tonsil tissue sections by ChipCytometry using a combination of CD3, CD8, Vα7.2, CD161, PLZF, and CD19 markers (Fig. 5, Supplemental Fig. 4). MAIT cells were identified as CD3^+^Vα7.2^+^CD161^+^PLZF^+^ cells. In (Fig. 5, all MAIT cells were CD8^+^ and were located within the T cell zone and adjacent to the B cell zone (indicated by white arrows). In Supplemental Fig. 4, we found CD8^+^ as well as CD8^−^ MAIT cells (a few examples are indicated by white arrows) side by side with another relatively rare tonsillar innate-like T cell subset, the γδ-T cells (white arrows with asterisk). Interestingly, MAIT as well as γδ-T cells were not only found within the T cell zone in the second example, but a few also adjacent and within the CD19^+^ area.

### Identification and phenotyping of human tonsillar MAIT cells by cell suspension ChipCytometry

**FIGURE 6.**
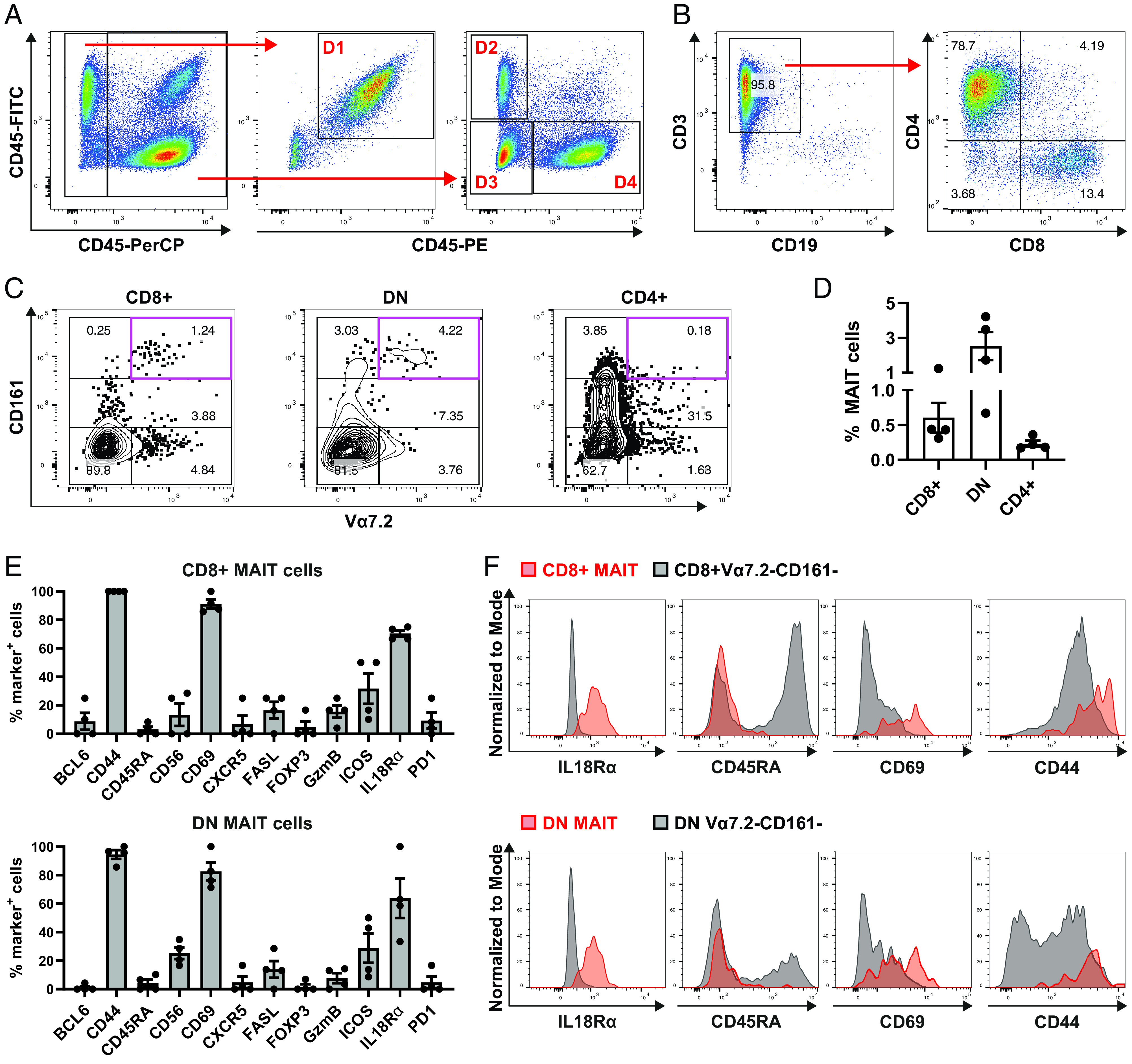
Identification and phenotyping of tonsillar MAIT cells using cell suspension ChipCytometry. Cell suspensions of four human tonsils were enriched for CD3^+^ cells and analyzed by ChipCytometry with a panel of 19 fluorochrome-labeled Abs. (**A**) Pooled samples were de-barcoded to identify different donors. Barcodes consisted of combinations of CD45 Abs that were labeled with different fluorochromes. Donor 1 (D1) = PerCP^−^FITC^+^PE^+^; donor 2 (D2) = PerCP^+^FITC^+^PE^−^, donor 3 (D3) = PerCP^+^FITC^−^PE^−^, and donor 4 (D4) = PerCP^+^FITC^−^PE^+^. (**B**) The following gating strategy was applied to identify MAIT cells: first, we gated on CD3^+^CD19^−^ cells. Within this gate, subpopulations according to CD4 and CD8 expression were defined, including CD4^+^, CD8^+^, and DN T cells. (**C**) Within each T cell subset, MAIT cells were identified by Vα7.2 and CD161^++^ expression. (**D**) Enumeration of MAIT cells within T cell subsets. (**E**) Phenotype of CD8^+^ and DN MAIT cells. (**F**) Representative plots of IL-18Rα, CD45RA, CD69, and CD44 expression by CD8^+^ and DN MAIT cells (red) compared with Vα7.2^−^CD161^−^ conventional T cells within CD8^+^ and DN T cells, respectively.

Besides the identification of CD161^++^/MAIT cells within CD3-enriched tonsil-derived single-cell suspensions via unsupervised clustering (Fig. 3B, 3C, Supplemental Fig. 3), we also identified and phenotyped MAIT cells by a traditional manual gating approach using the same dataset (Fig. 6A–F). After de-barcoding of the different donors (Fig. 6A), MAIT cells were identified using the following gating strategy (Fig. 6B, 6C): first, we gated on CD19^−^CD3^+^ T cells, and within this gate CD4^+^, CD8^+^, and DN T cell subsets were identified. Within each of those subsets, MAIT cells were defined as Vα7.2^+^CD161^++^, and their frequencies were determined. The MAIT cell frequencies within the CD8^+^ T cells were similar to the frequencies observed in the flow cytometry experiment (Fig. 4A, 4B). Using ChipCytometry, we identified ∼6-fold higher frequency of tonsillar DN MAIT cells than tonsillar CD8^+^ MAIT cells (Fig. 6D); however, we did not observe any considerable frequencies of CD4^+^ MAIT cells. By determining the frequencies of MAIT cells expressing different phenotyping markers, we found that CD8^+^ and DN MAIT cells shared a similar phenotype (Fig. 6E, 6F). Most MAIT cells were CD69^+^, indicating an activated phenotype. In addition, most MAIT cells were CD45RA^−^CD44^+^, indicating a memory and Ag-experienced phenotype. MAIT cells also expressed high levels of IL-18Rα, and ∼30% of MAIT cells expressed the costimulatory receptor ICOS. There was a very small fraction of FASL^+^ and GZMB^+^ MAIT cells, however. MAIT cells were considered negative for BCL6, CXCR5, PD1, and FOXP3. In summary, we found that tonsillar MAIT cells have an activated memory phenotype (CD44^+^CD45RA^−^CD69^+^ICOS^+^) and express typical innate-associated markers such as IL-18Rα. This correlates well with the generally accepted phenotype of MAIT cells described in the literature (40–42).

## Discussion

We have demonstrated, using the palatine tonsil as a test case, that ChipCytometry is a viable method that combines the power of traditional cytometry with the spatial information available from tissue sections. Using this method, we were able to demonstrate the immune cell compartments of the palatine tonsil, including the B cell zone with its subcompartments of naive (IgD^+^CD10^−^) and germinal center (IgD^−^CD10^+^) B cells and the T cell zone (Fig. 1, Supplemental Figs. 1A, 2A). Within the CD3^+^ T cell compartment, we could identify multiple T cell subsets, for example CD4^+^PD1^+^ T cells, most of which were located within the CD19^+^ B cell zone, with a few cells present within the T cell zone (Fig. 2, Supplemental Figs. 1B, 2B). We performed more stringent phenotyping of tonsillar T cells using cell suspension ChipCytometry and identified two subtypes of CD4^+^PD1^+^ cells including pre-TFH (CD4^+^PD1^+^CXCR5^−^BCL6^mid^) and TFH (CD4^+^PD1^+^CXCR5^+^BCL6^high^) cells, which were distinct from non-TFH (CD4^+^PD1^−^CXCR5^−^BCL6^low^) cells (Fig. 3E). TFH cells are a specialized immune subset that is crucial for forming protective immunity by providing T cell help to B cells (17, 19). We were able to demonstrate that ChipCytometry is sufficiently sensitive to identify very rare palatine tonsillar immune subsets such as the innate-like T cell subset MAIT cells. We identified and located MAIT cells in tissue sections (Fig. 5, Supplemental Fig. 4) and could further phenotype them using single-cell suspension ChipCytometry (Fig. 6). We confirmed that tonsillar MAIT cells have an activated, memory-like, and innate-like phenotype similar to that seen in blood-derived MAIT cells. Additionally, we identified a second innate-like T cell subset, the γδ-T cells, distributed alongside MAIT cells and located predominately in the T cell zone but with a few cells scattered within the CD19^+^ B cell zone (Fig. 5, Supplemental Fig. 4). Besides manual and supervised identification of compartments and cell types (Figs. 3E, 6), we successfully performed unsupervised clustering using FlowSOM to identify different cell types that are included in the cell suspension ChipCytometry dataset (Fig. 3A–D), which is an analysis strategy similar to what has been used for similar data obtained from other methods such as mass cytometry (35, 39). We identified 12 cell groups within the CD3^+^ tonsillar T cells, including expected cell types such as pre-TFH/TFH cells and CD161^++^/MAIT cells. The grouping of Vα7.2^−^CD161^++^ cells with MAIT cells in one cluster confirms that they share a similar phenotype (40). The FlowSOM algorithm partitioned some cell types based on the heterogenous expression of a single marker (e.g., CD44 within CD4^+^CD45RA^−^ cells or GZMB within CD8^+^CD45RA^−^ cells). Clustering into fewer, but potentially biologically more meaningful cell groups can be achieved by performing manual merging of similar clusters, as suggested by Nowicka et al. (39). Direct FlowSOM clustering into a smaller number of clusters can be problematic. First, cluster stability could suffer, and second, there is the danger that not all expected and rare cell types would be detected (39).

The examples above outline the advantages of ChipCytometry in which multiplex tissue cytometry data, including phenotyping and spatial information from intact tissue, can be combined with cell suspension data that include phenotyping information on a single-cell level. Multiple adjacent positions of a tissue section can be scanned and stitched together to obtain a relatively large image for analysis (theoretically up to a limit of 1 × 2 cm^2^) (Supplemental Fig. 2). In addition, the method allows users to acquire a theoretically unlimited number of markers on a single sample (6).

The ChipCytometry technique uses directly labeled primary Abs that are commercially widely available for flow cytometry. Panel design is straightforward, as no or little compensation is required. Imaging for each fluorochrome in an Ab mixture is performed sequentially for each fluorochrome at each position. Samples are preserved after analysis and can be stored up to 20 mo without significant biomarker degradation (7). This allows reanalysis of samples even after project completion or sample collection and processing and storage on different sites with subsequent centralized analysis.

As ChipCytometry includes two imaging steps, pre- and poststaining, some correction can be made for autofluorescence by the subtraction of the background from the poststaining image. Further advantages of ChipCytometry in comparison with alternative methods include a greater resolution limit of 500 nm as opposed to 1000 nm for imaging mass cytometry, and wider dynamic range (eight decades versus two to three decades in conventional fluorescence microscopy). ChipCytometry also has a higher scannable area than imaging mass cytometry (1 × 2 cm^2^ versus 1 mm^2^) (5, 6).

There are, however, some limitations to the ChipCytometry technique. This method is a serial and iterative process in which Abs from each subsequent round of staining are left in place and the signal is removed by photobleaching. In theory, issues due to steric hindrance could, therefore, be anticipated. Despite the highly multiplexed nature of ChipCytometry, we have not yet encountered any issues due to steric hindrance beyond any well-known Ab pairs from other applications such as flow cytometry or mass cytometry. An example is steric hindrance between the Ab pair Vδ2 clone B6 and γδTCR clone 11F2 (43).

Another factor is the negative impact of repeated photobleaching cycles on Ag integrity, most likely caused by photodamage of proteins that can absorb high-energy light in the UV region (44). A device upgrade from the manufacturer consisting of a longpass filter that blocks damaging UV in the white light filter module during the photobleaching process was provided to mitigate the destructive effect of repeated photobleaching cycles. Further mitigation can be accomplished by limiting the use of dyes to fluorochromes with a higher excitation wavelength (FITC, PE, and PerCP) and photobleaching only through their respective filters instead of using the universal white light filter module for photobleaching. Although these precautions generally minimize the destructive effect of repeated photobleaching, particular sensitive epitopes need to be stained early in the process. This requires, however, that each new epitope is tested to determine its relative sensitivity before its consideration in panel design. Sensitive markers applied in the panels of this study are indicated with an asterisk in Tables I–III.

Because of the optimization required, we find that ChipCytometry has a relatively slow sample throughput. In addition, each position on a sample chip is scanned one after another, and staining is performed in a serial iterative process. Sample throughput of cell suspensions can be increased using barcodes, which allows simultaneous analysis of multiple pooled samples as shown in (Fig. 6A. This approach limits, however, the maximum cell number per sample. Alternatively, sample throughput can no doubt be increased substantially through the use of automated ChipCytometry systems.

In summary, ChipCytometry is a viable and versatile method for immunophenotyping for both tissue sections as well as cell suspensions. We were able to show the general palatine tonsil architecture including immune compartments and localize and phenotype immune subsets, including very rare immune cell types. ChipCytometry overcomes some technical and practical method–specific limitations of other similar methods, although it has a relatively low throughput and requires substantial stain-specific optimization. This approach does lend itself to specific applications in immunology research through multistain imaging of specific immune subsets, which require multiple markers for definition, especially when applied in parallel with current high-content analysis of cell suspensions and single-cell RNA sequencing approaches.

## Supplementary Material

Data Supplement
