# Defining T-cell subsets in human tonsils using Chipcytometry

**DOI:** 10.4049/jimmunol.2100063

**Published:** 2021-06-07

**Authors:** Joachim P Hagel, Kyle Bennett, Francesca Buffa, Paul Klenerman, Christian B Willberg, Kate Powell

**Affiliations:** * Peter Medawar Building for Pathogen Research, Nuffield Department of Medicine, University of Oxford, Oxford OX1 3SY, United Kingdom; † Computational Biology and Integrative Genomics, Department of Oncology, University of Oxford, Oxford OX3 7DQ, United Kingdom; ‡ NIHR Biomedical Research Centre, John Radcliffe Hospital, Oxford University Hospitals NHS Foundation Trust, Oxford OX3 9DU, United Kingdom; § Translational Gastroenterology Unit, Nuffield Department of Medicine, University of Oxford, Oxford OX3 9DU, United Kingdom

## Abstract

Chipcytometry is a multiplex imaging method that can be used to analyse either cell suspensions or tissue sections. Images are acquired by iterative cycles of immunostaining with fluorescently labelled antibodies followed by photobleaching, which allows the accumulation of multiple markers on a single sample. In this study we explored the feasibility of using Chipcytometry to identify and phenotype cell subsets, including rare cell types, using a combination of tissue sections and single-cell suspensions. Using Chipcytometry of tissue sections, we successfully demonstrated the architecture of human palatine tonsils, including the B- and T-cell zones, and characterised sub-compartments such as the B-cell mantle and germinal centre zone, as well as intra-follicular PD1-expressing CD4+ T cells. Additionally, we were able to identify the rare tonsillar T-cell subsets, MAIT and γδ-T cells, within tonsil tissue. Using single-cell suspension Chipcytometry, we further dissected human tonsillar T-cell subsets via unsupervised clustering analysis as well as supervised traditional manual gating. We were able to show that PD1+CD4+ T cells are comprised of CXCR5+BCL6high TFH cells and CXCR5-BCL6mid pre-TFH cells. Both supervised and unsupervised analysis approaches identified MAIT cells in single cell suspensions, confirming a phenotype similar to that of blood-derived MAIT cells. Here, we demonstrate that Chipcytometry is a viable method for single-cell suspension cytometry and analysis, with the additional benefit of allowing phenotyping in a spatial context using tissue sections.

## Introduction

Many novel technologies have recently emerged for multi-parameter cytometry analysis of cells and tissues, offering the exciting prospect of visualising cellular phenotyping within a spatial context. These methods utilise a variety of immunostaining methodologies applied to fluorescence microscopy or mass-spectrometry imaging techniques to acquire information on the expression of multiple markers. Each method has its own advantages and disadvantages. For example, the number of markers that can be assessed on a single sample, the sample processing time and throughput, the commercial availability of reagents, operating costs and system purchasing costs are system-specific (1–5).

Chipcytometry is an imaging-based multiplex immunophenotyping method where samples, either cell suspensions or tissue sections, are immobilised on slides inside a microfluidic chip (6). Through consecutive and iterative cycles of staining with fluorophore-labelled antibodies, imaging and subsequent photo-bleaching, a theoretically unlimited number of markers can be measured on a single sample. Advantages of Chipcytometry over other methods include that samples are preserved after analysis and can be stored for at least 20 months without significant degradation (7). This allows re-analysis with additional markers, or sample collection and storage at different sites with subsequent centralised analysis. In addition, the development of a panel of markers is relatively straightforward using standard flow cytometry antibodies. Combinations of up to five fluorophores can be used for each cycle.

To explore the feasibility of using Chipcytometry for immune-phenotyping we employed lymphoid palatine tonsil tissue as it is very rich in immune cells and has a well-established and distinct architecture. Palatine tonsils are secondary lymphoid tissues that belong to the Waldeyer’s ring which further includes the adenoids, tubal and lingual tonsils (8, 9). They are located at the transition between the mouth and oropharynx and are a part of the mucosal-associated lymphoid tissues (10, 11). The tonsils are covered by an epithelium that increases the tonsil surface by forming crypts, in which specialised cells can take up antigen and transport it to subepithelial areas where it can get in contact with immune cells (10, 12). The immune cells are organised in two major zones within the tonsils: B cell follicles, and the extrafollicular region in which T cells and B cells become activated. Lymphoid follicles contain B cells and are therefore characterised by CD19+ expression. Primary lymphoid follicles do not have a germinal centre (13). Secondary lymphoid follicles consist of a mantle zone including naive (IgD+CD10-) B cells; they also contain a germinal centre including germinal centre (IgD-CD10+) B cells (14, 15). Germinal centres are places of B cell proliferation, differentiation and maturation (13). Besides B cells, lymphoid follicles also contain a network of stromal-derived follicular dendritic cells, which are important for antigen presentation to B cells (16). Additionally, B cell follicles contain T cells that are critical for the humoral immune response (17). The extrafollicular region (also called the T-cell zone) contains T cells which are primarily CD4+ T cells with fewer CD8+ T cells. Besides T cells, the extrafollicular region includes interdigitating dendritic cells, macrophages and high-endothelial venules. The latter are specialised venules that allow circulating lymphocytes to enter the lymphoid tissue (13).

Tonsillar CD4+ T-cell subsets can be divided into 3 major groups (18). Most intrafollicular T cells belong to the follicular B-helper T cell group (TFH, CD4+PD1+CXCR5+BCL6high) (17, 18). B cell and TFH interactions are crucial for the formation of the humoral immune response and support B cell somatic hypermutation and antibody class switching (17, 19). Other tonsillar CD4+ T-cell subsets include non-TFH (CD4+PD1-CXCR5-BCL6low) and pre-TFH (CD4+PD1+CXCR5-BCL6mid). Pre-TFH cells develop from activated antigen-specific CD4+ T cells. The transcriptional program that is initiated in pre-TFH cells can induce CXCR5 expression enabling the cells to migrate towards the B-cell follicle (18, 20).

Most CD4+ and CD8+ T cells that enter palatine tonsils are naive cells competing for antigen in order to reach maturity (10, 13), but there are also unconventional T-cell subsets found within the tonsil, for example innate-like T cells including MAIT (mucosal-associated invariant T cells) and γδ-T cells (21–23). MAIT cells are memory-type and antigen-experienced cells with peripheral tissue homing properties (24, 25). They are characterised by their semi-invariant T-cell receptor recognising microbial-derived riboflavin metabolites presented by a conserved MHC-Class I-like molecule called MR1 (26, 27). MAIT cells can, however, also be activated in a TCR-independent manner by cytokines such as IL12, IL18 and IL15 (28, 29). Most MAIT cells are CD8+ or DN (double negative) in blood. They can be identified by tetramer staining or using a combination of markers including Vα7.2 and CD161 (30, 31). MAIT cells also express the innate-like marker CD218a (IL18Rα) and the transcription factor PLZF (31). MAIT-cell selection and development begins in the thymus and MAIT cells are thought to acquire their memory antigen-experienced phenotype via contact with microbes in the periphery (32). MAIT-cell frequencies in tonsil are approximately 10 times lower than in blood (21). MAIT-cell responses play multiple roles in infection, inflammation and cancer (29, 33, 34).

In order to establish the utility of the Chipcytometry technique, using both cell suspensions and tissue sections, we aimed to depict the general tonsil architecture, showing different tonsillar regions and sub-compartments. We localised and phenotyped immune subsets including follicular CD4+ T-cell subsets and rarer immune cells such as MAIT cells and finally analysed data obtained from Chipcytometry of cell suspensions for more stringent phenotyping of immune subsets.

## Material and Methods

### 1 Tissues and cells

Human tonsillar tissue was obtained from routine tonsillectomies collected by the Translational Gastroenterology Unit (TGU) Biobank, John Radcliffe Hospital, Oxford, following informed consent under approved study protocol 16/YH/O247. Tissue sections were prepared by snap freezing samples in OCT cutting medium (OCT embedding matrix, CellPath) and cryosectioning onto glass coverslips. Single cell suspensions were prepared by mechanical disruption of tonsil samples. Human blood samples were obtained from the NHSBT (National Health Service Blood and Transplant) John Radcliffe Hospital, Oxford. PBMCs were isolated from on a Lymphoprep gradient (STEMCELL Technologies) according to manufacturer’s instructions.

### 2 Chipcytometry of cell suspensions

Tonsil-derived CD3+ T cells were isolated by positive selection from single cell suspensions using magnetic CD3-Microbeads (Miltenyi) and treated with Human TruStain FcX (BioLegend) to block unwanted Fc Receptor-mediated staining. Cells from different donors were barcoded with anti-CD45 antibodies (pan-leucocyte marker) coupled to FITC, PE or PerCP-Cy5.5 to allow analysis of multiple donors on a single chip. Barcodes consisted of different single anti-CD45 stains, or combinations of differently labelled anti-CD45 antibodies. The CXCR5 antibody does not recognise fixed epitope and therefore staining was performed simultaneously with CD45 barcode staining for 5 minutes at 4 °C. Donor-derived cells were mixed and loaded into cell suspension chips (ZellSafe™ Cells – Chips, Zellkraftwerk, Germany), allowed to adhere to the chip surface for 10 minutes at room temperature, then non-adhered cells were washed off with PBS. The initial acquisition, performed with the chip cytometer (Zellscanner ONE, Zellkraftwerk, Germany), included barcodes and CXCR5 stain. Samples were fixed (fixation buffer, Zellkraftwerk, Germany) for 5 minutes at room temperature and subsequent markers were acquired in iterative rounds of photo-bleaching, staining and imaging. FITC-, PE- or PerCP/PerCP-Cy5.5-coupled antibodies were applied in mixes (up to 3 per round). Surface antibodies were incubated for 10 minutes at room temperature. For intracellular markers, True-Nuclear™ Transcription Factor Buffer Set (BioLegend) was used as follows: chips were rinsed and incubated with permeabilisation buffer for 1 h followed by rinsing and incubation with fixation buffer for a further hour before finally washing with permeabilisation buffer. Antibody mixes for intracellular staining were prepared in permeabilization buffer and incubated for 30 minutes (all permeabilisation and staining steps performed at room temperature). The following 19 markers were applied to the samples: BCL6, CD3, CD4, CD8, CD19, CD44, CD45, CD45RA, CD56, CD69, CD161, CXCR5, FASL, FOXP3, Granzyme B (GZMB), ICOS, IL18Rα (CD218a), PD1, Vα7.2. For antibody details please see **Table I** and **II**.

### 3 Chipcytometry of tissue sections

Cryosections were cut directly onto polylysine or APES-coated coverslips (Sigma-Aldrich) and fixed immediately using freshly prepared 0.1M phosphate buffered 4% paraformaldehyde (Sigma-Aldrich) or Zellkraftwerk fixation buffer (Zellkraftwerk, Germany) for 10 minutes at room temperature. After washing in PBS, sections on coverslips were assembled into tissue chips (Zellsafe™ Tissue – Chips, Zellkraftwerk, Germany) which were immediately filled with PBS or storage buffer (Zellkraftwerk, Germany). Before use, the storage buffer was washed out of the chip with approximately 5 ml of PBS. Tonsil sections were initially blocked by incubating in 3% normal goat serum with 2% BSA (Thermo Fisher) in PBS for at least one hour at room temperature. Immunostaining was performed at room temperature for 30 minutes using 0.5 ml of diluted antibody solution per chip. Antibody cocktails were diluted in PBS alone or PBS containing 2% BSA or 2% normal goat serum. A total of 12 markers were acquired in iterative rounds of photo-bleaching, staining and imaging: CD3, CD4, CD8, CD10, CD19, CD161, γδTCR, IgD, PD1, PLZF, Vα7.2 and histone H3 (not shown). For antibody details please see **Table III**.

### 4 Flow cytometry

Cells were stained with a viability dye (Live/Dead fixable near-IR dead cell stain kit, Invitrogen) and fluorochrome-labelled antibodies for 30 minutes at 4°C. The following antibodies were used: CD3-eFluor450 (OKT3, eBioscience), CD8-VioGreen (BW135/80, Miltenyi), γδTCR-FITC (5A6.E9, Invitrogen), CD161-PE and CD161-APC (191B8, Miltenyi), Vδ2-PerCP-Cy5.5 (B6, BioLegend), CD56-PE-Cy7 (HCD56, BioLegend), Vα7.2-APC and Vα7.2-PerCP-Cy5.5 (3C10, BioLegend), CCR7-FITC (G043H7), CD62L-PE (DREG-56, BioLegend). Data was acquired by a MACSQuant Analyzer 10 (Miltenyi).

### 5 Data processing and statistical methods

Flow cytometry and Chipcytometry data was analysed using FlowJo (Versions 10.6.2 and 10.7.1, Beckton Dickinson) and bar charts were generated using GraphPad Prism software (Version 8.4.3). For supervised manual gating analysis of flow cytometry and Chipcytometry data, biexponential transformation was applied in FlowJo. For unsupervised clustering analysis, Chipcytometry cell suspension data was first transformed and pre-processed in FlowJo and then exported to R (RStudio Version 1.2.5042, R Version 3.6.2). The data were transformed using HyperLog transformation. The parameters of this transformation were adjusted and rescaling was performed so that for each marker the distribution spanned the whole dynamic range. The transformed data was binned into integer histogram channels ranging from 0 to 1023. Outliers and fluorescence anomalies outside the axis limits after transformation were assigned to either the minimum or maximum value. All datapoints with an assigned value of 0 or 1023 after the transformation were removed from further analysis. An exception was made for CXCR5, which was transformed using logarithmic transformation, because suitable rescaling could not be achieved with HyperLog in FlowJo. Data was pre-processed by excluding CD19+ cells and gating on CD3+ cells in FlowJo. FlowSOM R package (Version 1.18.0) (35) was used to identify cell clusters within the data set using all markers except for CD45, which was used for barcoding, and CD3 and CD19, as these were used for gating during pre-processing. Parameter chosen were: SOM grid = 10 x 10, seed = 1234. The resulting SOM nodes were used in the R package ConsensusClusterPlus (1.50.0) to obtain the final optimal clusters (36). A delta area plot was created with a maximum of k = 20 metaclusters, which indicated cluster stability at a metacluster number of k = 12. Cluster information for 12 meta clusters was exported and data was further analysed in FlowJo for visualisation, including t-SNE analysis (Fit-SNE, iteration: 3000, perplexity: 50, KNN algorithm: Exact, learning rate: Auto (opt-SNE)). For cluster characterisation, the distribution of the intensity of each marker was considered in each cluster and the median value taken [median marker expression]. This was displayed as heatmap using pheatmap package (Version 1.0.12). In addition to comparing median marker expression directly, Marker Enrichment Modeling was performed for cluster characterisation using the R package MEM (Version 2.0.0) (37, 38).

### 6 Image processing

Images for publication were downloaded from the chip cytometer as 16-bit uncompressed Tiff files and converted to 8-bit per channel RGB TIFF files in ImageJ/Fiji (version1.5). Scale bars were added in Adobe Photoshop CS4 according to the metadata stored with the image. Images were cropped, annotations (arrows, text etc.) were added, and brightness and contrast adjusted as necessary in Photoshop. Files were exported as flattened 300 dpi TIFF files with LZW compression. Files of each image processing stage were saved.

## Results

### Defining tonsillar architecture in humans using tissue Chipcytometry

**Figure 1:**
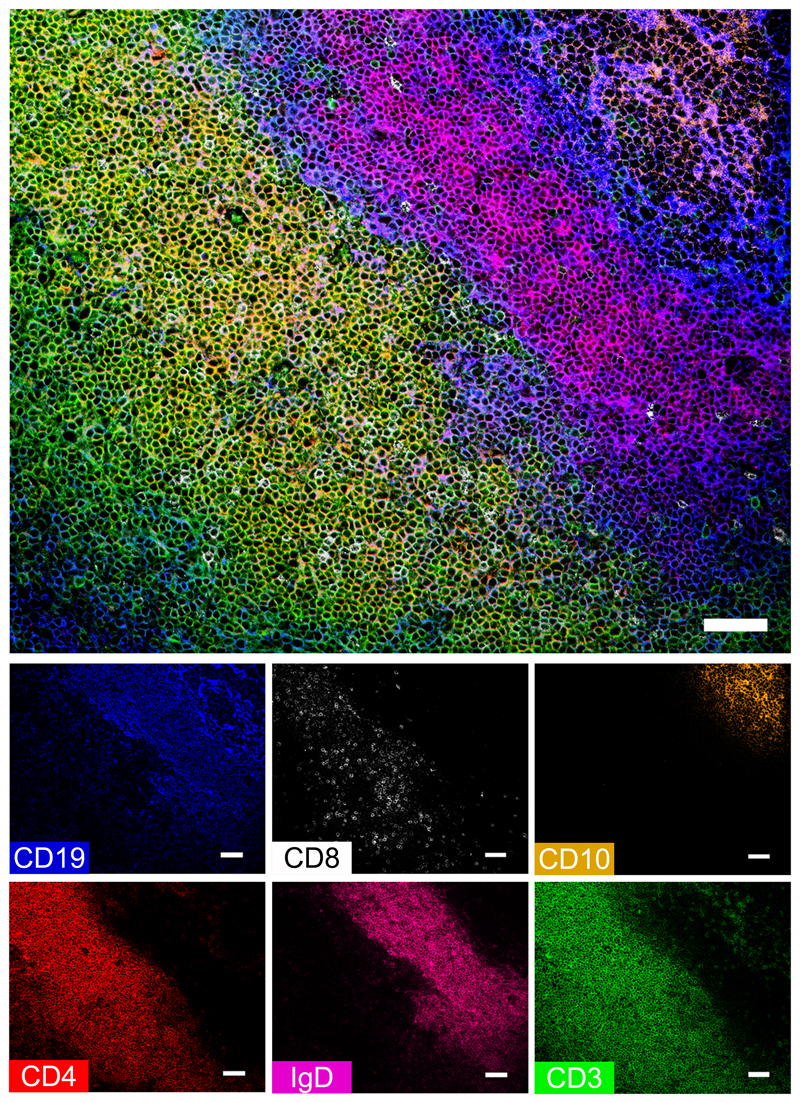
Human tonsil architecture resolved using Chipcytometry. Sections of paraformaldehyde-fixed human tonsil tissue mounted in microfluidic chips were immunostained in consecutive runs using a panel of 12 fluorochrome-labelled antibodies. The top image was compiled by selecting six markers; CD19 (blue), CD8 (white), CD10 (orange), CD4 (red), IgD (magenta) and CD3 (green), assigning colours arbitrarily from the lookup table and merging to form a composite image. Expression of individual markers is shown in the small panels. The B-cell zone (B-cell follicle) is characterised by CD19 expression, in which IgD outlines the mantle zone containing IgD-expressing naive B cells, and CD10 outlines the germinal centre containing CD10+ germinal centre B cells. Adjacent to the B-cell zone, CD3 expression outlines the T-cell zone (extrafollicular zone), including CD4+ and CD8+ T cells. Scale bars represent 50 μm.

A panel of 12 antibodies was employed to establish the anatomy of the palatine tonsil by Chipcytometry. A combination of T-cell markers (CD3, CD4, CD8) and B-cell markers (CD19, CD10, IgD) were chosen to depict general palatine tonsil architecture (Figure 1, Suppl. Figure 1a and 2a). Specifically, the lymphoid follicle zone (B-cell zone) was defined by CD19 expression and could be further characterised by the differential expression of IgD and CD10. Naive B cells (IgD+CD10-) are mainly located in the B-cell mantle zone, and germinal centre B cells (IgD-CD10+) are located in the B-cell germinal centre zone (14, 15). The extrafollicular zone (T-cell zone) adjacent to the B-cell zone was defined by CD3 expression. CD3+ T cells included CD4-expressing and CD8-expressing cells. A few CD3+ T cells could also be found within the B-cell zone.

### Identification of CD4 T-cell subsets in human tonsils

**Figure 2:**
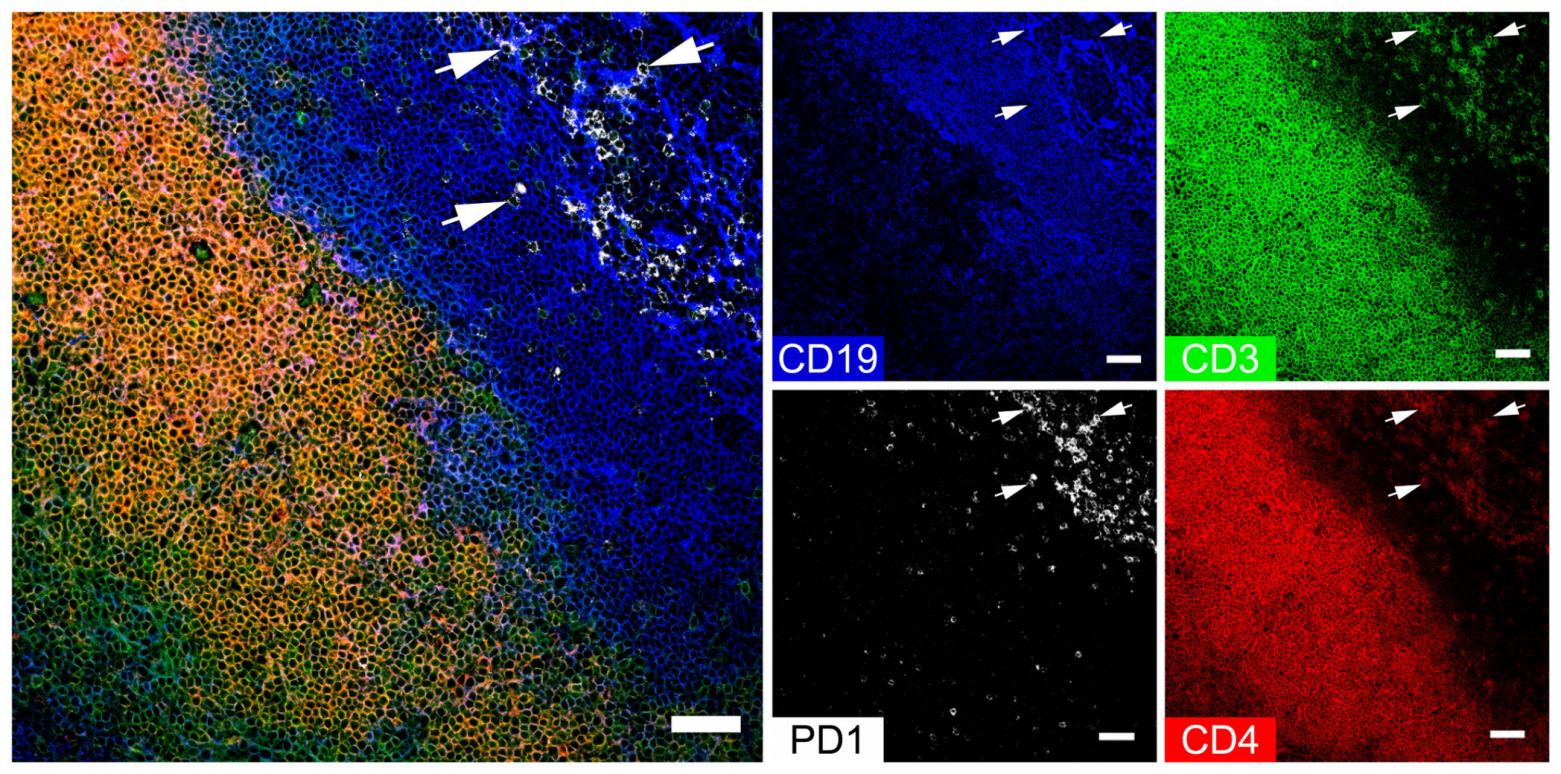
Identification of CD4+ T-cell subsets in human tonsils. A combination of CD4, CD19, CD3 and PD1 were chosen from the full marker panel to identify CD4+ T cells in the tonsil. The large multicolour composite image shows a merged image of CD19 (blue), CD3 (green), PD1 (white) and CD4 (red), while the smaller images demonstrate the expression of each marker individually. The T-cell zone is characterised by CD3 expression and includes CD4+ T cells; CD3+CD4+ cells appear yellow / orange in combination. PD1-expressing CD3+CD4+ are predominantly located in the B-cell zone, but a few are also located in the T-cell zone. This subset most likely includes pre-follicular (pre-TFH) and follicular T-helper cells (TFH). CD3+CD4+PD1-cells are predominantly located in the extrafollicular region and include non-follicular T helper cells (non-TFH). White arrowheads indicate examples of CD3+CD4+PD1+ cells. Scale bars represent 50 μm.

From the full panel of 12 markers, a combination of CD3, CD4, PD1 and CD19 staining was chosen to identify palatine tonsillar T-cell subsets expressing PD1 (Figure 2, Suppl. Figure 1b and 2b). CD3 expression was concentrated within the extrafollicular T-cell zone; however, we were also able to identify some intrafollicular CD3+ T cells. Many of those T cells were CD4+ and PD1+. A few rare CD4+PD1+ T cells could also be found within the extrafollicular region (T-cell zone). Tonsillar CD3+CD4+PD1+ T cells most likely include Pre-TFH and TFH subsets (18) as opposed to CD3+CD4+PD1- cells that most likely include non-TFH cells. Examples of some of the many CD3+CD4+PD1+ T cells are indicated in Figure 2 and Suppl. Figure 1bs by white arrowheads.

### Identification and phenotyping of tonsillar CD3 T-cell subsets by cell suspension Chipcytometry

**Figure 3:**
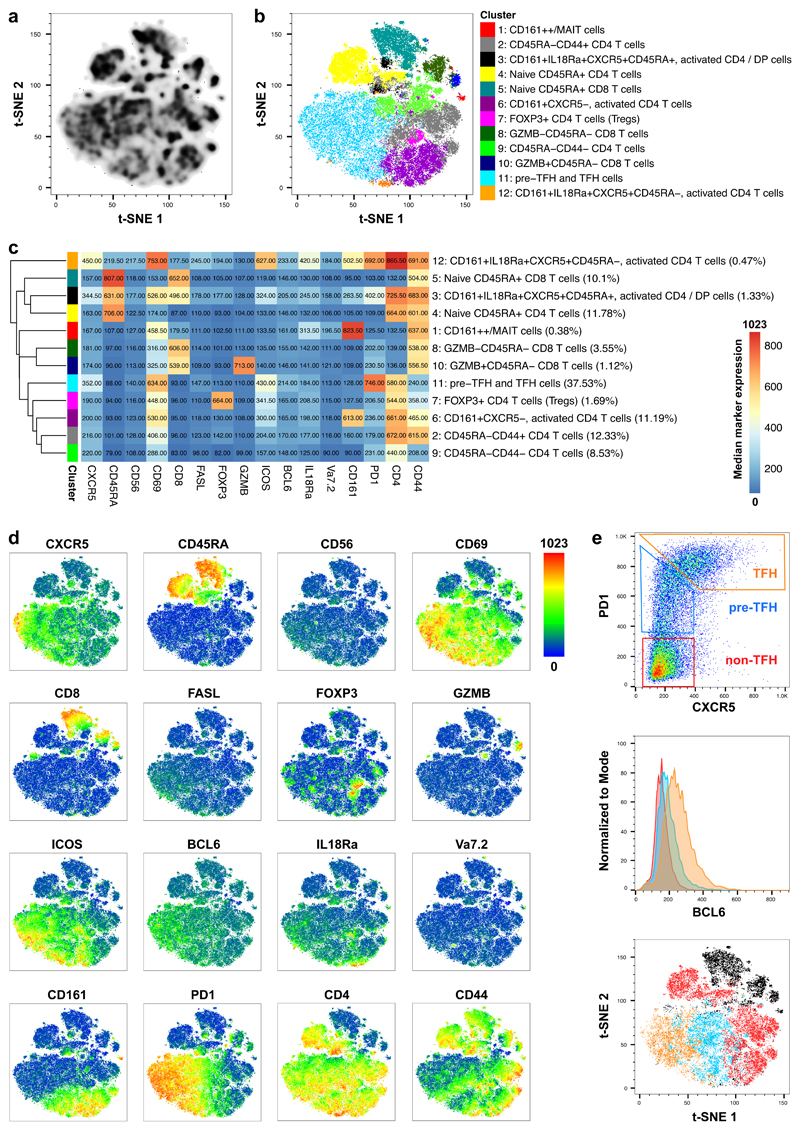
Phenotyping of tonsil-derived CD3-enriched cells by cell suspension Chipcytometry. Four human tonsils were processed to single cell suspensions, positively enriched for CD3+ T cells, barcoded, loaded onto a microfluidic chip, fixed and subsequently analysed by Chipcytometry with a panel of 19 fluorochrome-labelled antibodies. (**a**) Density plot shows t-SNE analysis of concatenated samples including all CD3+CD19-cells. (**b**) Clusters that were identified via FlowSOM were projected onto the t-SNE coordinates and color-coded as indicated. (**c**) A heatmap showing median marker intensities within each cell cluster was generated to characterise cell clusters and derive cell labels. The heatmap colour represents median marker expression across all 4 aggregated samples. (**d**) T-SNE plots show the expression of 16 markers within the aggregated samples. (**e**) Top panel: Within CD4+ T cells, the subsets TFH, pre-TFH and non-TFH cells were identified according to expression of CXCR5 and PD1. Centre panel: BCL6 expression is shown within non-TFH (red), pre-TFH (blue) and TFH (orange) T-cell subsets in human tonsils. Bottom panel: T-SNE plot shows non-TFH (red), pre-TFH (blue), TFH (orange) and non-CD4 T cells (black). Plots in (e) are derived from one donor and are representative of 4 donors.

To identify and phenotype tonsillar CD3 T-cell subsets, cell suspensions produced by dissociation of human palatine tonsil tissue were enriched for CD3+ T cells (N=4). Cells from each donor were barcoded with CD45-antibodies coupled to different fluorochromes, allowing individual donors to be distinguished in the pooled samples. A staining panel consisting of the following 19 markers was used: BCL6, CD3, CD4, CD8, CD19, CD44, CD45, CD45RA, CD56, CD69, CD161, CXCR5, FASL, FOXP3, Granzyme B (GZMB), ICOS, IL18Rα (CD218a), PD1 and Vα7.2. T-cell subsets were identified via unsupervised clustering using FlowSOM, a method based on Self-Organising-Map clustering (35, 39), and visualised using t-SNE (Figure 3a). ConsensusClusterPlus was used to estimate the optimal number of clusters within the data set, and FlowSOM clustering was performed into 12 groups (Figure 3b). As the process for labelling cell clusters is not established, we applied a layered approach for cluster labelling using median marker expression together with marker enrichment modeling in a semi-supervised manner in addition to gating in FlowJo (Figure 3c, Suppl. Figure 3a, b). The identified CD3+ T-cell subsets included various CD4+ T-cell subsets, CD8+ T-cell subsets and unconventional, innate-like T-cell subsets such as MAIT cells. We found one naive CD4+CD45RA+ T-cell subset (cluster 4) and two CD4+CD45RA- subsets which differed in regard to CD44 expression (cluster 2 = CD44+, cluster 9 = CD44-). Each of these subsets comprised about 10% of total T cells. Additionally, we found three subsets that contained CD4+ T cells expressing CD161, which is a marker generally associated with innate-like and memory cell types (40). Those were CD161+CXCR5-CD69+ICOS+CD45RA- (cluster 6, about 10% of total T cells), CD161+CXCR5+IL18Rα+CD69+PD1+ICOS+CD45RA- (cluster 12, 0.47% of total T cells), and CD161+CXCR5+IL18Rα+CD69+PD1+ICOS+CD45RA+ (cluster 3, 1.33% of total T cells). The latter included besides CD4+ T cells also a substantial fraction of double-positive T cells and showed lower levels of CD161, CXCR5, IL18Rα, CD69, PD1 and ICOS expression compared to cluster 12. Other CD4+ T-cell subsets included FOXP3+ cells, which represent the T-reg population (cluster 7, 1.69% of total T cells) and pre-follicular / follicular CD4 T-helper cells (cluster 11, CD4+CXCR5+/-PD1+ICOS+CD69+) which comprised 37.53% of total T cells. In addition to CD4 T-cell subsets, we found a naive CD8+CD45RA+ subset (cluster 5, approx. 10% of total T cells) and two CD8+CD45RA- subsets, of which one was GZMB+ (cluster 10, 1.12% of total T cells) and the other was GZMB- (cluster 8, 3.55% of total T cells), respectively. We also found a cluster that contained CD161++ T cells including MAIT cells (CD161++Vα7.2+/-IL18Rα+CD69+, cluster 1, 0.38% of total T cells), which were either CD8+ or double-negative (DN). The expression of the 16 phenotyping markers that were used for clustering are displayed on t-SNE plots in Figure 3d.

We further analysed the data set via manual gating, to elaborate on the characterisation of PD1-expressing and follicular CD4 T-cell subsets. We stratified CD4+ T cells according to PD1 and CXCR5 expression (Figure 3e) as previously shown by Thornhill et al. (18). The subsets included CXCR5+PD1+ TFH, CXCR5-PD1+ pre-TFH and CXCR5-PD1- non-TFH cells. These cell types differed in BCL6 expression; TFH cells had the highest levels of BCL6 expression, pre-TFH intermediate levels of BCL6 expression, and non-TFH the lowest levels of BCL6 expression. These three CD4 subsets also segregated on the t-SNE plot. With our characterisation of tonsil-derived CD4 T cell subsets, were able to replicate results previously described in the literature by Thornhill et al. (18).

### Identifying rarer T-cell subsets in tonsils by tissue Chipcytometry including MAIT cells and γδ-T cells

**Figure 4:**
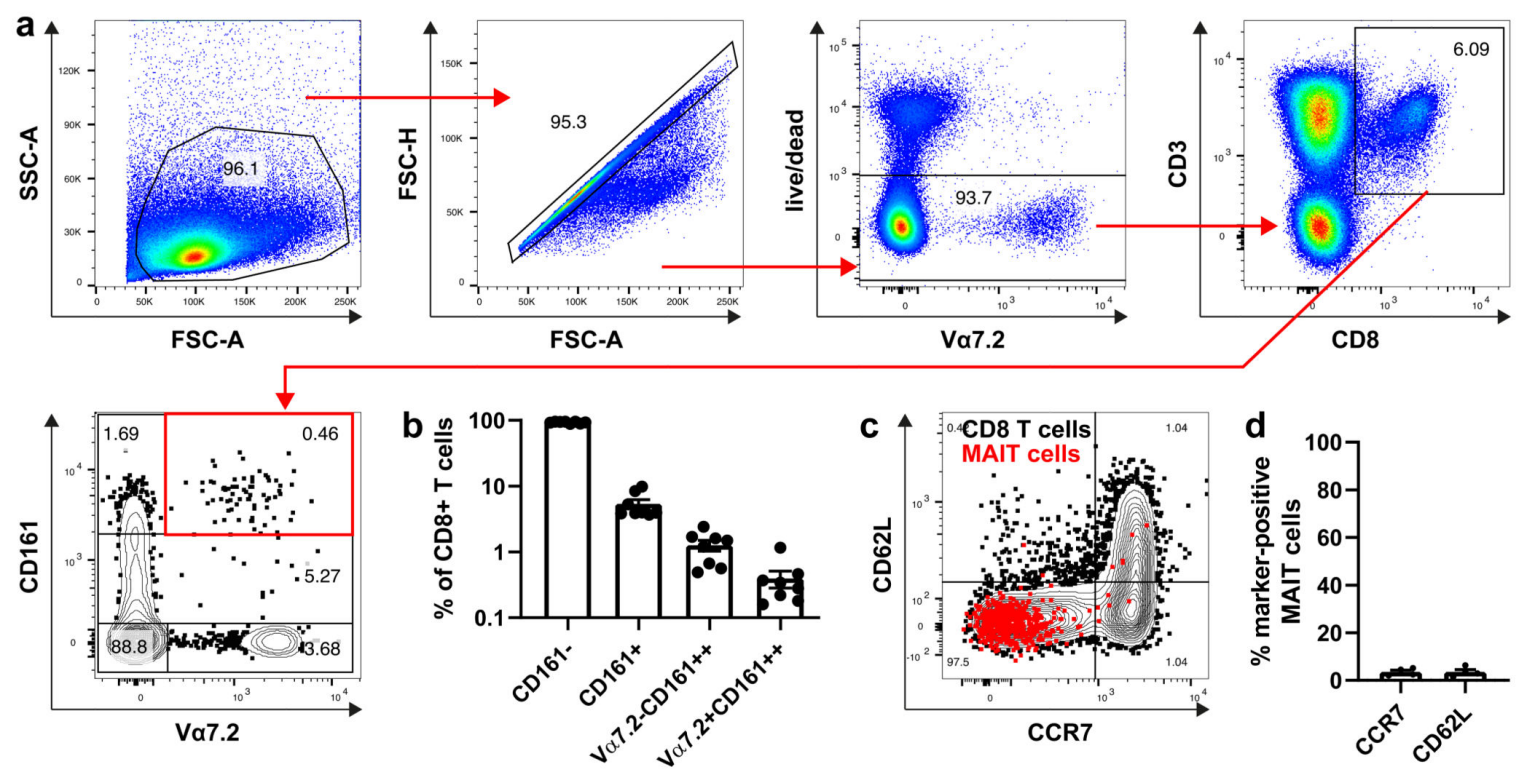
Flow cytometry identifies CD8+ MAIT cells as a rare subset within human tonsils. Human tonsils were processed to single cell suspensions and analysed by flow cytometry. Stains included LIVE/DEAD™ Fixable dye, CD3, CD8, Vα7.2, CD161, CCR7 and CD62L. (**a**) Gating strategy is shown to identify CD8+ MAIT cells (Vα7.2+CD161++) within tonsils. (**b**) Enumeration of CD8 T-cell subsets (8 tonsils from 6 donors). (**c**) CCR7 and CD62 expression of total CD8 T cells (black) and MAIT cells (red). The flow cytometry plot is representative of 4 biological replicates. (**d**) Enumeration of CCR7 and CD62L expressing MAIT cells (N=4).

Next, we aimed to identify and phenotype rare cell subsets within palatine tonsillar tissue. MAIT cells have been described as a rare cell subset within human palatine tonsil tissue (21, 22). We could confirm this by the analysis of 8 palatine tonsils of 6 different donors using flow cytometry (Figure 4a-b). MAIT cells were defined as CD3+CD8+Vα7.2+CD161++. The frequency of MAIT cells ranged from approximately 0.1 to 1 % of total CD8 T cells. This is 10-fold lower compared to blood (41). Interestingly, palatine tonsillar MAIT cells did not show typical lymphoid tissue homing marker expression CCR7 and CD62L (Figure 4c-d).

**Figure 5:**
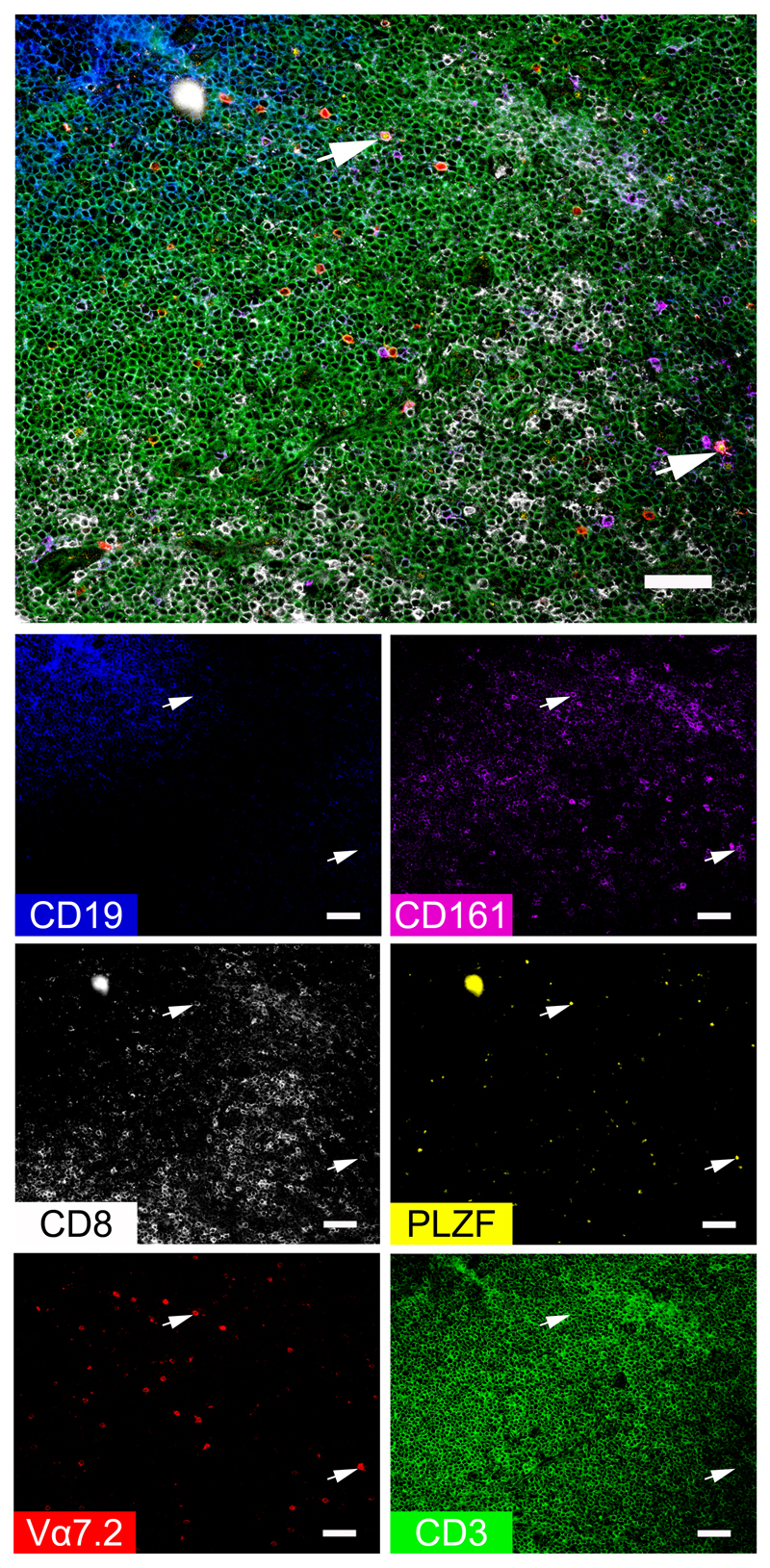
Identification of rare immune subsets in human tonsils. A combination of Vα7.2 (shown in red), CD8 (white), CD161 (magenta), PLZF (yellow), CD3 (green) and CD19 (blue) markers were selected from the available panel to illustrate the presence of MAIT cells within the tonsil. Single-colour images demonstrate the expression of each marker individually, while the larger image shows a merged composite of all six markers. White arrowheads indicate examples of cells that co-express CD3, CD8, Vα7.2, CD161 and PLZF, which represent CD8+ MAIT cells. During the iterative staining process dust particles can be introduced into the microfluidic chip, generating autofluorescencent artefacts such as those seen here at the top left of the CD8, PLZF and merged images. Scale bars represent 50 μm.

We were able to identify CD8+ MAIT cells in palatine tonsil tissue sections by Chipcytometry using a combination of CD3, CD8, Vα7.2, CD161, PLZF and CD19 markers (Figure 5, Suppl. Figure 4). MAIT cells were identified as CD3+Vα7.2+CD161+PLZF+ cells. In Figure 5, all MAIT cells were CD8+ and were located within the T-cell zone and adjacent to the B-cell zone (indicated by white arrows). In Suppl. Figure 4, we found CD8+ as well as CD8- MAIT cells (a few examples are indicated by white arrows) side by side with another relatively rare tonsillar innate-like T-cell subset, the γδ-T cells (white arrows with asterisk). Interestingly, MAIT as well as γδ-T cells were not only found within the T-cell zone in the second example, but a few also adjacent and within the CD19+ area.

### Identification and phenotyping of human tonsillar MAIT cells by cell suspension Chipcytometry

**Figure 6:**
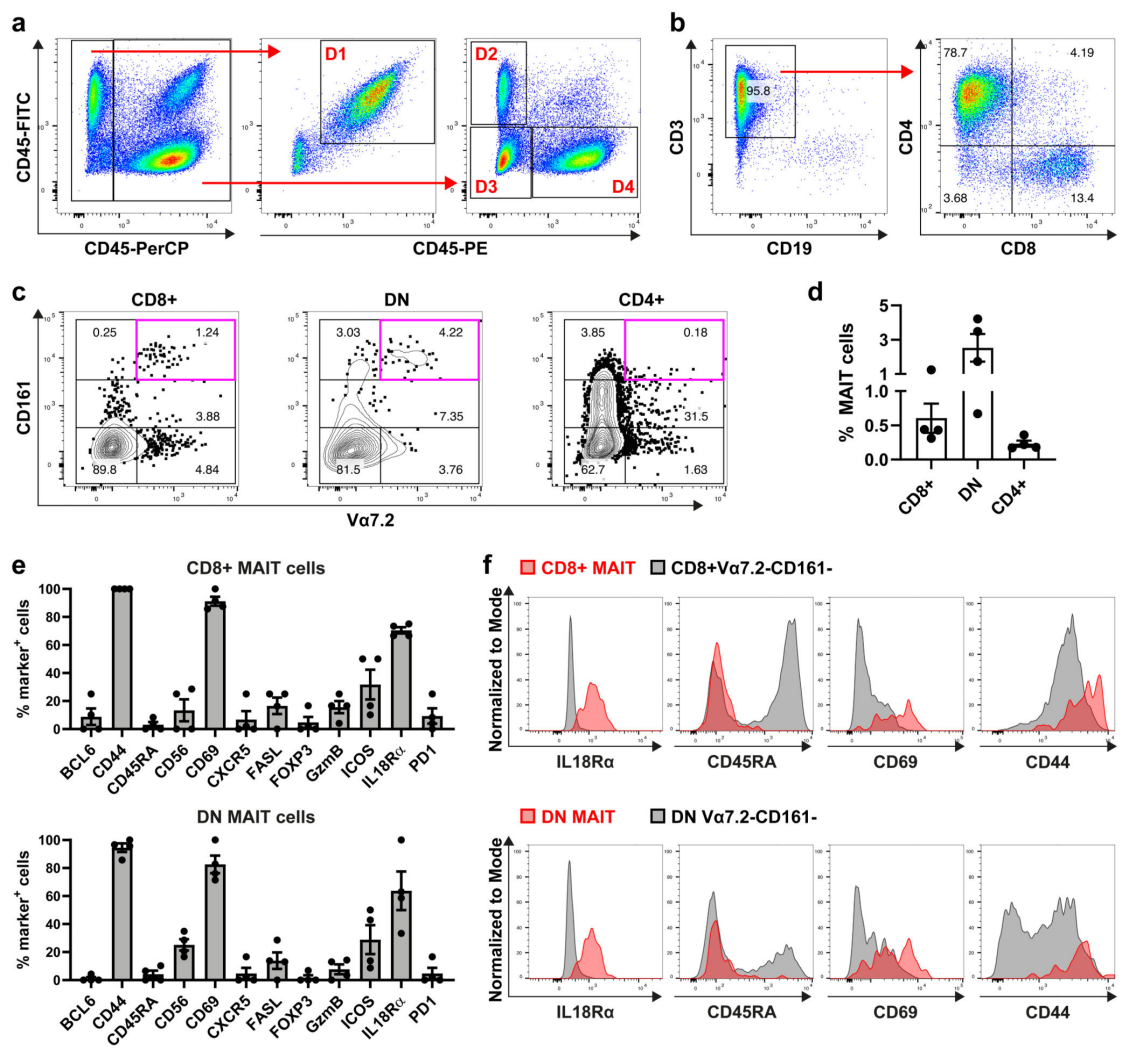
Identification and phenotyping of tonsillar MAIT cells using cell suspension Chipcytometry. Cell suspensions of four human tonsils were enriched for CD3+ cells and analysed by Chipcytometry with a panel of 19 fluorochrome-labelled antibodies. (**a**) Pooled samples were de-barcoded to identify different donors. Barcodes consisted of combinations of CD45 antibodies that were labelled with different fluorochromes. Donor 1 (D1) = PerCP-FITC+PE+, Donor 2 (D2) = PerCP+FITC+PE-, Donor 3 (D3) = PerCP+FITC-PE-, Donor 4 (D4) = PerCP+FITC-PE+. (**b**) The following gating strategy was applied to identify MAIT cells: First, we gated on CD3+CD19-cells. Within this gate, subpopulations according to CD4 and CD8 expression were defined, including CD4+, CD8+ and DN (double negative) T cells. (**c**) Within each T-cell subset, MAIT cells were identified by Vα7.2 and CD161++ expression. (**d**) Enumeration of MAIT cells within T-cell subsets. (**e**) Phenotype of CD8+ and DN MAIT cells. (**f**) Representative plots of IL18Rα, CD45RA, CD69 and CD44 expression by CD8+ and DN MAIT cells (red) compared to Vα7.2-CD161-conventional T cells within CD8+ and DN MAIT cells, respectively.

Besides the identification of CD161++/MAIT cells within CD3-enriched tonsil-derived single cell suspensions via unsupervised clustering (Figure 3b-c, Suppl. Figure 3), we also identified and phenotyped MAIT cells by a traditional manual gating approach using the same data set (Figure 6a-f). After de-barcoding of the different donors (Figure 6a), MAIT cells were identified using the following gating strategy (Figure 6b, c): First, we gated on CD19-CD3+ T cells, and within this gate CD4+, CD8+ and DN T-cell subsets were identified. Within each of those subsets, MAIT cells were defined as Vα7.2+CD161++ and their frequencies were determined. The MAIT-cell frequencies within the CD8+ T cells was similar to the frequencies observed in the flow cytometry experiment (Figure 4a-b). Using Chipcytometry we identified approximately 6-fold higher frequency of tonsillar DN MAIT cells than tonsillar CD8+ MAIT cells (Figure 6d), however, we did not observe any considerable frequencies of CD4+ MAIT cells. By determining the frequencies of MAIT cells expressing different phenotyping markers, we found that CD8+ and DN MAIT cells shared a similar phenotype (Figure 6e-f). Most MAIT cells were CD69+, indicating an activated phenotype. In addition, most MAIT cells were CD45RA-CD44+, indicating a memory-and antigen-experienced phenotype. MAIT cells also expressed high levels of IL18Rα, and about 30% of MAIT cells expressed the co-stimulatory receptor ICOS. There was a very small fraction of FASL+ and GZMB+ MAIT cells, however. MAIT cells were considered negative for BCL6, CXCR5, PD1 and FOXP3. In summary, we found that tonsillar MAIT cells have an activated memory phenotype (CD44+CD45RA-CD69+ICOS+) and express typical innate-associated markers such as IL18Rα. This correlates well with the generally accepted phenotype of MAIT cells described in the literature (40–42).

## Discussion

We have demonstrated, using the palatine tonsil as a test case, that Chipcytometry is a viable method which combines the power of traditional cytometry with the spatial information available from tissue sections. Using this method, we were able to demonstrate the immune cell compartments of the palatine tonsil, including the B-cell zone with its sub-compartments of naive (IgD+CD10-) and germinal centre (IgD-CD10+) B cells, and the T-cell zone (Figure 1, Suppl. Figure 1a and 2a). Within the CD3+ T-cell compartment we could identify multiple T-cell subsets, for example CD4+PD1+ T cells, most of which were located within the CD19+ B-cell zone, with a few cells present within the T-cell zone (Figure 2, Suppl. Figure 1b and 2b). We performed more stringent phenotyping of tonsillar T cells using cell suspension Chipcytometry, and identified two subtypes of CD4+PD1+ cells including pre-TFH (CD4+PD1+CXCR5-BCL6mid) and TFH (CD4+PD1+CXCR5+BCL6high) cells, which were distinct from non-TFH (CD4+PD1-CXCR5-BCL6low) cells (Figure 3e). TFH cells are a specialised immune subset that is crucial for forming protective immunity by providing T-cell help to B cells. We were able to demonstrate that Chipcytometry is sufficiently sensitive to identify very rare palatine tonsillar immune subsets such as the innate-like T-cell subset MAIT cells. We identified and located MAIT cells in tissue sections (Figure 5, Suppl. Figure 4) and could further phenotype them using single cell suspension Chipcytometry (Figure 6). We confirmed tonsillar MAIT cells have a similar activated, memory-like and innate-like phenotype to that seen in blood-derived MAIT cells. Additionally, we identified a second innate-like T-cell subset, the γδ-T cells, distributed alongside MAIT cells and located predominately in the T-cell zone, but with a few cells scattered within the CD19+ B-cell zone (Figure 5, Suppl. Figure 4). Besides manual and supervised identification of compartments and cell types (Figure 3e, Figure 6) we successfully performed unsupervised clustering using FlowSOM to identify different cell types that are included in the cell suspension Chipcytometry data set (Figure 3a-d), which is an analysis strategy similar to what has been used for similar data obtained from other methods such as CyTOF (35, 39). We identified 12 cell groups within the CD3+ tonsillar T cells, including expected cell types such as pre-TFH/TFH cells and CD161++/MAIT cells. The grouping of Vα7.2-CD161++ cells with MAIT cells in one cluster confirms that they share a similar phenotype (40). The FlowSOM algorithm partitioned some cell types based on heterogenous expression of a single marker, e.g., CD44 within CD45RA-CD4+ cells or GZMB within CD45-CD8+ cells. Clustering into fewer, but potentially biologically more meaningful cell groups can be achieved by performing manual merging of similar clusters as suggested by Nowicka et al. (39). Direct FlowSOM clustering into a smaller number of clusters can be problematic. First, cluster stability could suffer and secondly, there is the danger that not all expected and rare cell types would be detected (39).

The examples above outline the advantages of Chipcytometry where multiplex tissue cytometry data, including phenotyping and spatial information from intact tissue, can be combined with cell suspension data that includes phenotyping information on a single cell level. Multiple adjacent positions of a tissue section can be scanned and stitched together to obtain a relatively large image for analysis (theoretically up to a limit of 1x2cm^2^) (Suppl. Figure 2). In addition, the method allows users to acquire a theoretically unlimited number of markers on a single sample (6).

The Chipcytometry technique uses directly labelled primary antibodies that are commercially widely available for flow cytometry. Panel design is straightforward as no or little compensation is required. Imaging for each fluorochrome in an antibody cocktail is performed sequentially for each fluorochrome at each position. Samples are preserved after analysis and can be stored up to 20 months without significant biomarker degradation (7). This allows re-analysis of samples even after project completion or sample collection, and processing and storage on different sites with subsequent centralised analysis.

As Chipcytometry includes two imaging steps, pre- and post-staining, some correction can be made for autofluorescence by subtraction of background from the post-staining image. Further advantages of Chipcytometry in comparison to alternative methods include a greater resolution limit of 500 nm as opposed to 1000 nm for imaging mass spectrometry, and wider dynamic range (8 decades vs 2-3 decades in conventional fluorescence microscopy). Chipcytometry also has a higher scannable area than imaging mass cytometry (1x2 cm^2^ vs 1 mm^2^) (5, 6).

There are, however, some limitations to the chip-cytometry technique. This method is a serial and iterative process, where antibodies from each subsequent round of staining are left in place and the signal removed by photobleaching. In theory, issues due to steric hindrance could, therefore, be anticipated. Despite the highly multiplexed nature of Chipcytometry, we have not yet encountered any issues due to steric hindrance beyond any well-known antibody pairs from other applications such as flow cytometry or CyTOF. An example is steric hindrance between the antibody pair Vδ2 clone B6 and γδTCR clone 11F2 (43).

Another factor is the impact of repeated photobleaching cycles on antigen integrity, most likely caused by photodamage of proteins which can absorb high-energy light in the UV region (44). A device upgrade from the manufacturer consisting of a longpass filter that blocks damaging UV in the white light filter module during the photobleaching process was provided to mitigate the destructive effect of repeated photobleaching cycles. Further mitigation can be accomplished by limiting the use of dyes to fluorochromes with a higher wavelength (FITC, PE, PerCP) and photobleaching only through their respective filters instead of using the universal white light filter module for photobleaching. While these precautions generally minimise the destructive effect of repeated photobleaching, particular sensitive epitopes need to be stained early in the process. This requires, however, that each new epitope is tested to determine its relative sensitivity before its consideration in panel design. Sensitive markers applied in the panels of this study are indicated with an asterisk in **Table I-III**.

Due to the optimisation required, we find that Chipcytometry has a relatively slow sample throughput. In addition, each position on a sample chip is scanned one after another, and staining is performed in a serial iterative process. Sample throughput can be increased using barcodes which allows simultaneous analysis of multiple pooled samples as shown in Figure 6a. This approach limits, however, the maximum cell number per sample. Alternatively, sample throughput can no doubt be increased substantially through the use of automated Chipcytometry systems.

In summary, Chipcytometry is a viable and versatile method for immunophenotyping for both tissue sections as well as cell suspensions. We were able to show the general palatine tonsil architecture including immune compartments and localise and phenotype immune subsets including very rare immune cell types. Chipcytometry overcomes some technical and practical method-specific limitations of other similar methods although it has a relatively low throughput and requires substantial stain-specific optimisation. This approach does lend itself to specific applications in immunology research through multi-stain imaging of specific immune subsets, which require multiple markers for definition, especially when applied in parallel with current high-content analysis of cell suspensions and scRNASeq approaches.

## Supplementary Material

1

## Key points

1. Chipcytometry was used for multiplex-immunophenotyping of human tonsils.
2. It is viable for high-content profiling of cell suspensions and tissue sections.
3. Chipcytometry can identify and localise cell populations and rare cell types.

## Abbreviations

DN T cells: double negative T cells
GZMB: Granzyme B
MAIT cells: mucosal-associated invariant T cells
MEM: Marker Enrichment Modeling
TFH: T follicular helper cells.
