# Supplementary material for: Defining T-cell subsets in human tonsils using Chipcytometry
Source: J Immunol. Author manuscript; Available in PMC 2021 Sep 24. (PMC8278278; doi:10.4049/jimmunol.2100063)
Supplement: 1 [file EMS123014-supplement-1.pdf]

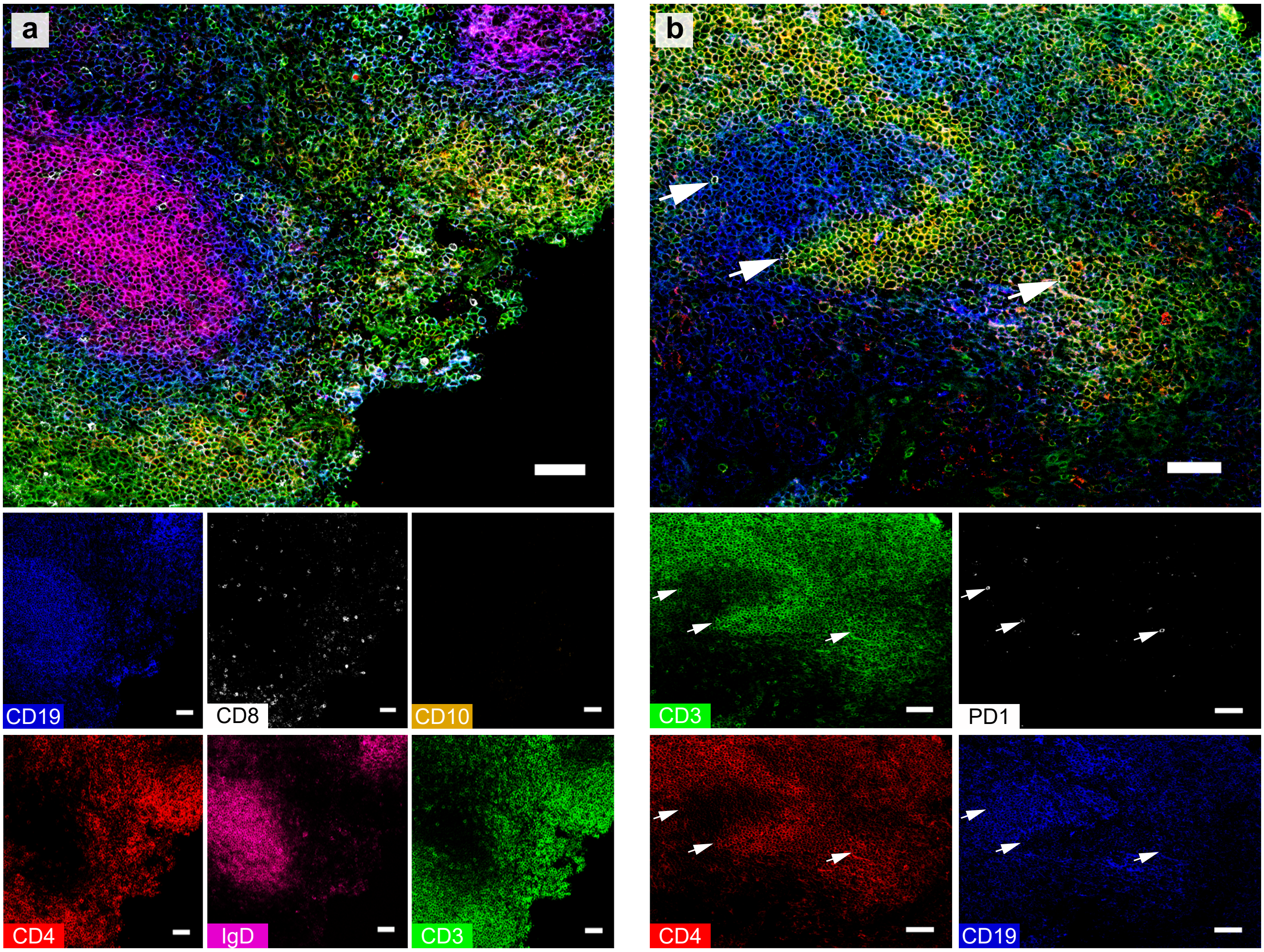


**Suppl. Figure 1: Identification of the general human tonsil architecture and tonsillar CD4+ T-cell subsets using Chipcytometry (second example).** Sections of paraformaldehyde-fixed human tonsil tissue mounted in microfluidic chips were immunostained in consecutive runs using a panel of 12 fluorochrome-labelled antibodies. (**a**) Six markers were selected to depict the general human tonsil architecture: CD19 (blue), CD8 (white), CD10 (orange), CD4 (red), IgD (magenta) and CD3 (green). The large multicolour image shows a composite image of all the 6 markers, whereas the expression of individual markers is shown in the small panels. The B-cell zone (B-cell follicle) is characterised by CD19 expression, in which IgD outlines the mantle zone containing IgD-expressing naive B cells, and CD10 outlines the germinal centre containing CD10+ germinal centre B cells. Adjacent to the B-cell zone, CD3 expression outlines the T-cell zone (extrafollicular zone), including CD4+ and CD8+ T cells. Scale bars represent 50 µm. (**b**) A combination of CD4, CD19, CD3 and PD1 was chosen from the full marker panel to identify tonsillar CD4+ T cells and their subsets according to PD1-expression. The large multicolour composite image shows a merged image of CD19 (blue), CD3 (green), PD1 (white) and CD4 (red), while the smaller images demonstrate the expression of each marker individually. The T-cell zone is characterised by CD3 expression and includes CD4+ T cells; CD3+CD4+ cells appear yellow / orange in combination. PD1-expressing CD3+CD4+ are predominantly located in the B-cell zone, but a few are also located in the T-cell zone. This subset most likely includes pre-follicular (pre-TFH) and follicular T-helper cells (TFH). CD3+CD4+PD1- cells are predominantly located in the extrafollicular region and include non-follicular T helper cells (non-TFH). White arrowheads indicate examples of CD3+CD4+PD1+ cells. Scale bars represent 50 µm.

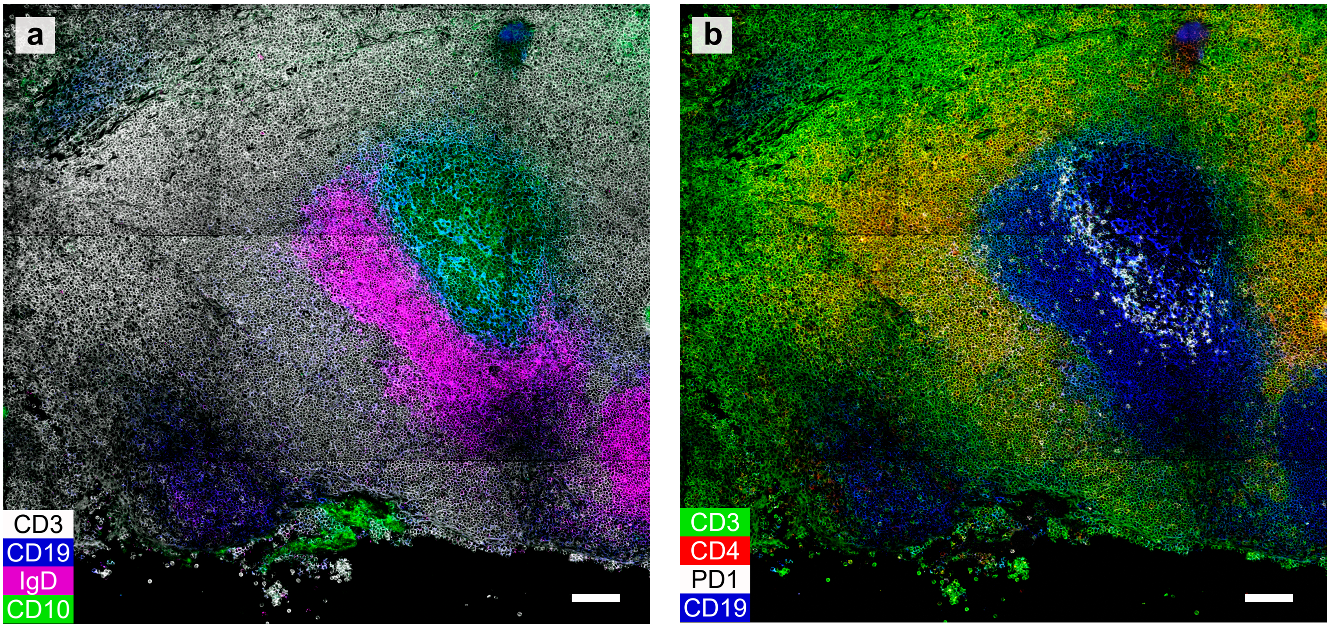


**Suppl. Figure 2: Chipcytometry allows the acquisition of multiple adjacent positions of a tissue section that can be tiled to obtain a larger field of view.** Human tonsil tissue was fixed, mounted on microfluidic chips and analysed by chip cytometry with panel of 12 fluorochrome-labelled antibodies. Multiple adjacent positions were recorded and stitched together to obtain an overview over a larger tissue area. Different combinations of markers were chosen to illustrate different immune compartments in the tissue. (**a**) CD19 (blue), CD10 (green), IgD (purple) and CD3 (white) were chosen to illustrate different tonsil regions including extrafollicular T-cell zone (CD3+), B cell follicle (CD19+) and B cell sub-compartments including mantle zone (IgD+CD10-) and germinal centre zone (IgD-CD10+). For further information please refer to **Figure 1** and **Suppl. Figure 1a**. (**b**) CD3 (green), CD4 (red), CD19 (blue) and PD1 (white) was chosen to depict PD1 expressing CD4 T cells and their location relative to B cell and T-cell zones. For further information refer to **Figure 2** and **Suppl. Figure 1b**. Scale bars represent 100 µm.

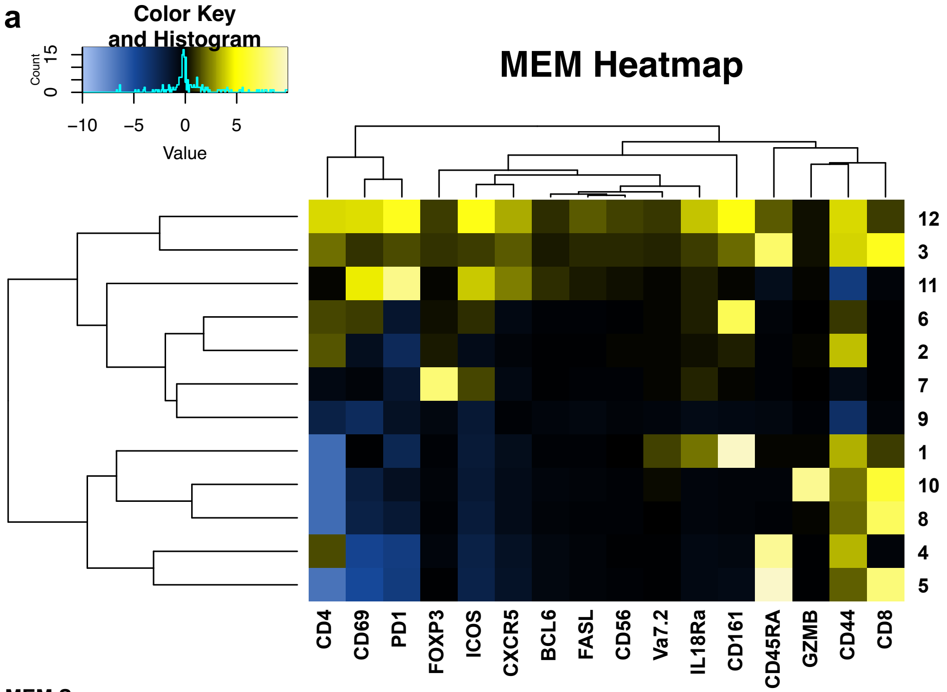


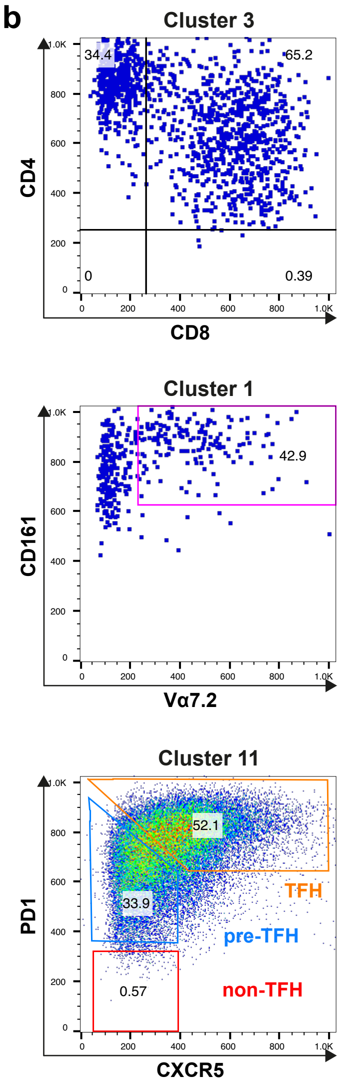


**<u>MEM Scores:</u>**

**Cluster 1:** ▲ CD161+10 CD44+3 IL18Ra+2 CD8+1 Va7.2+1 ▼ CD4-6 PD1-3 ICOS-2 CXCR5-1
**Cluster 2:** ▲ CD44+4 CD4+2 CD161+1 ▼ PD1-3 CD69-1 ICOS-1
**Cluster 3:** ▲ CD45RA+7 CD8+6 CD44+4 CXCR5+2 CD161+2 CD4+2 CD56+1 CD69+1 FASL+1 FOXP3+1 ICOS+1 IL18Ra+1 Va7.2+1 PD1+1 ▼ None
**Cluster 4:** ▲ CD45RA+9 CD44+3 CD4+1 ▼ CD69-5 PD1-4 ICOS-2 CXCR5-1 IL18Ra-1
**Cluster 5:** ▲ CD45RA+10 CD8+8 CD44+2 ▼ CD4-7 CD69-5 PD1-4 ICOS-2 CXCR5-1 IL18Ra-1 CD161-1
**Cluster 6:** ▲ CD161+7 CD69+1 ICOS+1 IL18Ra+1 CD4+1 CD44+1 ▼ PD1-2 CXCR5-1
**Cluster 7:** ▲ FOXP3+8 ICOS+1 IL18Ra+1 ▼ CXCR5-1 PD1-1 CD4-1 CD44-1
**Cluster 8:** ▲ CD8+7 CD44+2 ▼ CD4-6 CD69-2 ICOS-2 PD1-2 CXCR5-1
**Cluster 9:** ▲ None ▼ CD69-3 CD44-3 ICOS-2 CD4-2 IL18Ra-1 CD161-1 PD1-1
**Cluster 10:** ▲ GZMB+9 CD8+6 CD44+2 ▼ CD4-6 CD69-2 ICOS-2 CXCR5-1 PD1-1
**Cluster 11:** ▲ PD1+8 CD69+4 ICOS+4 CXCR5+2 BCL6+1 IL18Ra+1 ▼ CD44-4 CD45RA-1
**Cluster 12:** ▲ PD1+6 ICOS+5 CD161+5 CD69+4 IL18Ra+4 CD4+4 CD44+4 CXCR5+3 CD45RA+2 FASL+2 CD56+1 CD8+1 FOXP3+1 BCL6+1 Va7.2+1 ▼ None

**Suppl. Figure 3: Cluster characterisation using Marker Enrichment Modeling (MEM) and FlowJo gating.** (**a**) Cell clusters that were identified using FlowSOM as shown in **Figure 3b** were characterised with the help of Marker Enrichment Modeling in R as described by Diggins et al. (37, 38). MEM scores ranging from -10 to +10 indicate positive or negative enrichment of each marker within a population compared to the average of all other populations. (**b**) Examples of using FlowJo gating for cluster characterisation. Median marker expression (**Figure 3c**) and MEM scores (**Suppl. Figure 3a**) provided in certain cases ambiguous information for cell cluster labelling. An example is cluster 3, which showed increased median marker expression values and positive MEM scores for both CD8 and CD4 compared to the other clusters. To determine whether the cluster contained single positive CD4 and CD8 T cells or DP (double-positive) T cells, we inspected CD4 and CD8 expression in FlowJo within cluster 3 as shown in the top panel. This revealed that cluster 3 contained both single positive CD4 T cells and DP T cells, but no single positive CD8 T cells. Another example is cluster 1, for which FlowJo inspection (middle panel) revealed that it contained CD161++ T cells including both Vα7.2+ MAIT cells (pink gate) and Vα7.2- non-MAIT cells. The dichotomy of Vα7.2 expression in this cluster was not obvious from evaluating Vα7.2 median marker expression or MEM score. The last example of FlowJo cluster inspection (bottom panel) showed that cluster 11 contained both pre-TFH (CXCR5-PD1+) as well as TFH (CXCR5+PD1+) cells. Final labels for all cell clusters were determined in a multi-pronged approach considering median marker expression of each marker, MEM Scores and manual inspection of clusters in FlowJo.

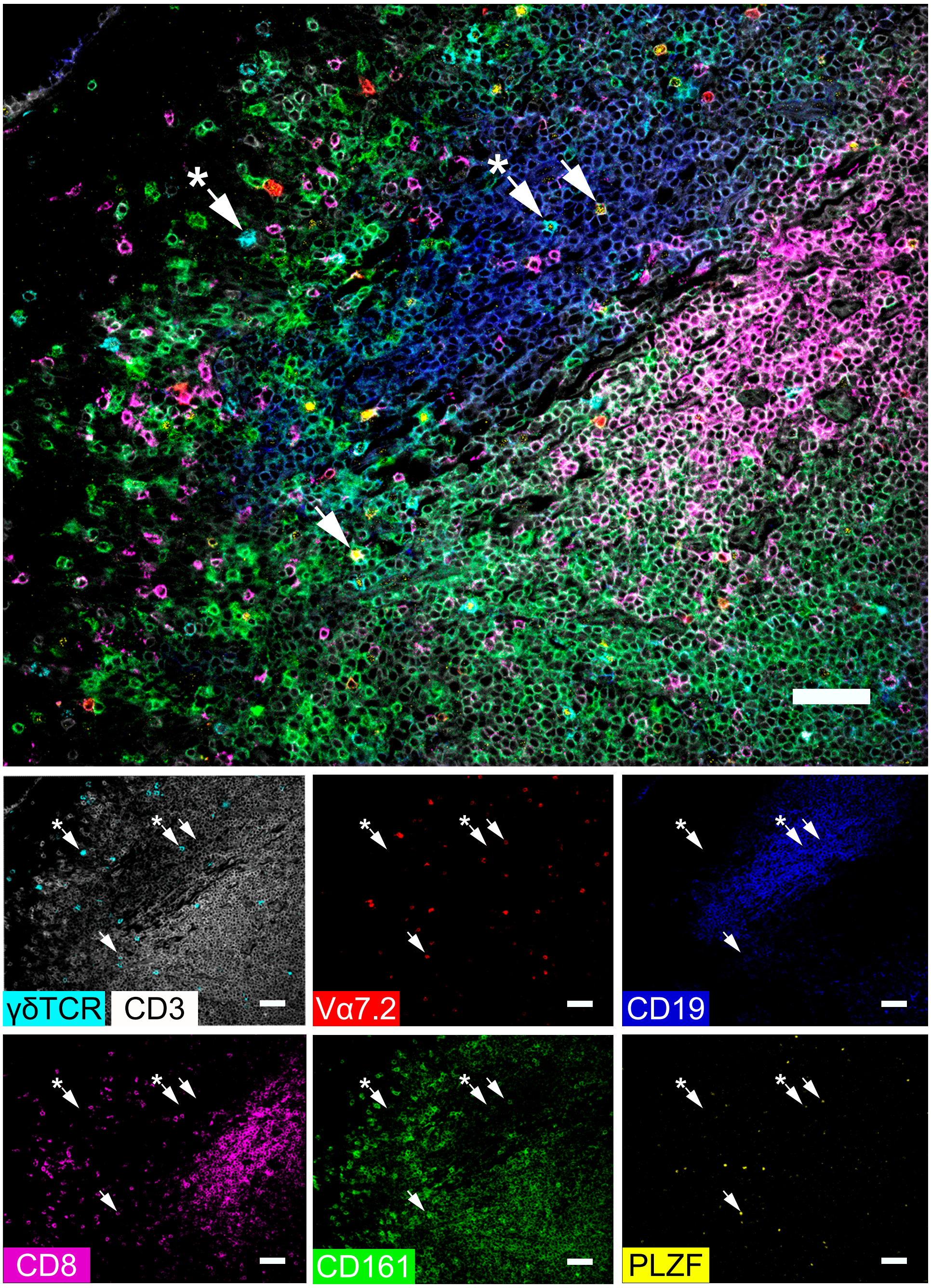


**Suppl. Figure 4: Identification of rare immune subsets in human tonsils including MAIT and γδ-T cells.** A combination of the markers γδ-TCR (cyan), CD3 (white), Vα7.2 (red), CD19 (blue), CD8 (magenta), CD161 (green) and PLZF (yellow) were selected from the available panel to illustrate the presence of MAIT cells and γδ-T cells within the tonsil. The large panel shows a merged multicolour composite image with all seven markers and the smaller panels show single- and dual-colour images as indicated. A few examples of MAIT cells (CD3+Vα7.2+CD161+PLZF+) are indicated by white arrowheads without an asterisk. CD8 was co-expressed in some of the MAIT cells, but there were also CD8- MAIT cells. γδ-T cells can be recognised side by side with MAIT cells in the T-cell zone (CD3+, white) but also adjacent and within the B cell zone (CD19+, blue). A few examples of many γδ-T cells are indicated by white arrows with an asterisk. Scale bars represent 50 μm.
